# Co-cultivation of primary porcine RPE cells and neuroretina induces inflammation: a potential inflammatory AMD-model

**DOI:** 10.1038/s41598-023-46029-8

**Published:** 2023-11-07

**Authors:** Agnes Fietz, Sven Schnichels, José Hurst

**Affiliations:** Centre for Ophthalmology, University Eye Hospital Tübingen, 72076 Tübingen, Germany

**Keywords:** Retinal diseases, Diseases of the nervous system

## Abstract

One common aspect in the pathology of many retinal diseases like age-related macular degeneration (AMD) is the death of retinal pigment epithelium (RPE) cells. RPE cells are essential for photoreceptor survival as they recycle and remove compounds of the visual cycle and secrete protective cytokines. Studying RPE cells is crucial to improve our understanding of retinal pathologies, yet only a few retinal ex vivo models include them or do so only indirectly. Besides the positive effects in indirect co-cultivation models, also a slight inflammation was observed. In this study we developed an ex vivo model consisting of a primary porcine RPE monolayer directly co-cultured with porcine retinal organ cultures, to investigate and simulate inflammatory retinal diseases, such as (dry) AMD. The direct co-cultivation resulted in immune reactivity (enhanced expression of pro-inflammatory cytokines e.g., *IL-1β*, *IL-6,* *IL-8*) and cell death. These effects were evaluated for the retinal explant as well as for the RPE-monolayer to further understand the complex interactions between these two compartments. Taken together, this ex vivo model can be used to study inflammatory retinal diseases like AMD as well as the rejection observed after RPE-transplantation.

## Introduction

Increasing numbers of ex vivo retinal damage models are being developed, to reduce animal use in experiments, and to simulate a variety of different retinopathies^1,2^. The pressure to avoid animal research has increased heavily over the last years, with the statement of the US environmental protection agency (US-EPA) to end animal testing by 2035. Therefore, there is a huge need for reliable, reproducible, and close-to-human alternative models. Numerous retinal organ models from different mammalian species were recently established^3–6^, for example ex vivo retinal models based on oxidative stress are used to investigate age related macular degeneration (AMD) or other retinal diseases like glaucoma^7–10^.

AMD is an acquired, chronic, macular disease characterized by progressive neurodegeneration of photoreceptors and retinal pigment epithelial (RPE) cells, leading to irreversible visual impairment. AMD is among the leading causes concerning central visual loss and legal blindness worldwide in people older than 60 years^11^. More than 8 million people may suffer from visual impairment due to AMD in the U.S., with a global prevalence of 170 million. Since AMD prevalence is directly linked to age, and as the global age has increased over the last decades by almost 10 years, the impact of AMD on the socio-economical life is expected to increase dramatically in the next years^11^. There are two major AMD forms: the early-stage, atrophic, non-exudative (also known as dry) form and the neovascular, exudative, type (also known as wet type). The neovascular form is typified by choroidal neovascularization in the sub retinal space and vascular leakage, as well as severe damage to the photoreceptor cells. In atrophic AMD, small deposits, called drusen, accumulate beneath the macula, resulting in degeneration of photoreceptors and RPE cells over time^12^. Most AMD cases start as the dry type and may progress to the wet type. Also, over 40% of advanced AMD cases, including both atrophic and neovascular AMD, are known as geographic atrophy (GA). GA can be seen as part of late-stage AMD and leads to central scotomas and permanent loss of visual acuity. The start and progression is triggered by intrinsic and extrinsic factors, e.g. high oxidative stress, photo-oxidation and environmental stressors like smoking. Also, mutations in several genes, mainly in the complement system, increase the risk of developing GA. After the development of drusen, lipofuscin accumulates (intermediate AMD), triggering inflammation via multiple pathways, ultimately resulting in photoreceptor and RPE loss, as well as in choriocapillaris and atrophic lesions that grow over time.

Despite its obvious detrimental effects to patients quality of life, there is still insufficient treatment or cure for the atrophic or geographic form. However, since 2022, there is a first therapeutic approach, which can slow down the degeneration of retinal cells when injected regularly^13^. In this study, central visual acuity is not improved by this therapeutic agent and the neovascular form occurs more frequently as a side effect^13^. Besides that, Zimura, a inhibitor of complement C, is now available in the US to treat geographic AMD with the aim to slow down the progress^14^. Similar, Syfovre, the first ever drug to treat atrophic AMD, was approved in the US in the beginning of 2023^13^.Nevertheless, despite the extensive research on AMD pathogenesis, the exact underlying mechanisms are yet to be elucidated.

Cultivation of ex vivo retinas mostly involve the use of pigs and rodents. Pigs are generally more suitable than rodents for studies on macular diseases (e.g., AMD), as pig retinae have a region called *area* *centralis* similar to the human macula, and the anatomy of porcine eyes closely resembles that of the human eye^15^. Thus, retinal organ cultures from porcine eyes are often used as ex vivo models^16^. An additional advantage of porcine retinal explants is their viability for at least eight days with only minimal degeneration^10,17,18^. Nevertheless, ex vivo retinal models also demonstrate disadvantages. The explanation causes the retina to detach from the underlying RPE, which has essential functions for the retinal survival. This tight, non-dividing monolayer resembles a barrier between the retina and the underlying choroid, and serve as "rubbish chutes" for the photoreceptors^19,20^. Furthermore, they have an important recycling role in the visual cycle and secrete various cytokines, like vascular endothelial growth factor (VEGF), which are essential for the survival of the retinal cells^21^. They also play an important role in immunosuppression and help to maintain the immune privilege^22,23^. Moreover, they take up shed photoreceptor outer segments (POS)^21^, therefore protecting photoreceptors against oxidative stress^24,25^. Impaired function or even loss of these cells is a hallmark of many retinal diseases such as AMD. RPE cells undergo various changes during aging, leading to the emergence of detectable accumulated extracellular material, drusen, between the RPE and the Bruch’s membrane. These deposits consist of components that attract microglia, resulting in the activation of resting microglia in the retina. This causes inflammation and degeneration of neighbouring photoreceptor cells, pathological processes that are part of the development of AMD. The fact that current retinal ex vivo models lack the RPE not only inadequately reflects the in vivo situation, but also misses a central aspect in the development of these diseases. Therefore, it is questionable to what extent experiments without RPE cells are meaningful. In order to improve the investigation of retinal disease pathogenesis, it is essential to include the RPE monolayer in previously established ex vivo damage models^26,27^. With that, investigations of the complex interactions between the RPE monolayer and the photoreceptors, as well of the exact pathogenesis of mentioned diseases, is possible.

Retinal explants and RPE cells can be indirectly co-cultured^27,28^. Indirect co-cultivation with primary, non-confluent, RPE cells demonstrates beneficial effects on the retina, like enhanced survival of photoreceptors and maintenance of synaptic vesicles^27,28^. On the other hand, slight immunoreactivity of the explant occurs after indirect co-cultivation^27^. In AMD-affected retinas, an increased immune reactivity was also observed, resulting in RPE cell death and photoreceptors over time^29^. As RPE cells can form a tight, functional monolayer in trans-well inserts^30^, we developed a direct co-cultivation model with a porcine retinal explant together with a functional, primary RPE monolayer^31^.

In this study, we investigated cell survival of the porcine retinal explant and the porcine primary RPE monolayer as well as the induction of the immune system. We were able to demonstrate that direct co-cultivation with primary porcine RPE monolayers led to the induction of an immune response as seen in increased expression of pro-inflammatory cytokines. The induced inflammation resulted in enhanced cell death in the retinal explant and demonstrated increased caspase 3/7-activity and TUNEL+ cells. Co-cultivation led also to a disruption and cell death of the tight RPE monolayer and enhanced deposit of neutral lipids. The seen inflammation was probably increased due to the high genetic variability of the RPE donors, when RPE monolayers were created. RPE transplantations, performed to treat AMD at early stages, involving xenogeneic or allogeneic sources, demonstrated a high risk of immunologic rejection^32^. Besides that, stem cell-derived RPE are also susceptible to immune rejections following transplantation^33^. Taken together, our direct porcine co-cultivation model can be used to mimic inflammation-driven retinal diseases like AMD in a short amount of time and can therefore be used to investigate new therapeutic options. Furthermore, immunologic rejections, as seen in RPE-transplantations, can be studied in detail.

## Results

### Co-cultivation led to inflammation in retinal explants as well as in RPE-monolayers

Current in vivo retinal co-culture models lack one important component: the RPE layer. To include this essential part, as a first step we established a functional primary, porcine RPE monolayer in trans-well Inserts^31^. These monolayers show high, in vivo like trans-epithelial-resistance (TER) values (> 250 Ωcm^2^), tight junctions in an in vivo like pattern (seen as Zonula occludens-1 (ZO-1) staining^31^) and protect the retina from oxidative stress^31^. To investigate the observed inflammatory effect in indirect co-cultivations^34^ further, primary porcine RPE monolayers were co-cultivated with porcine retinal explants for 24 h and 48 h and compared to retinal explants without RPE monolayer, which is referred as control group. Already after 24 h, a slight increase in pro-inflammatory cytokine expression was detectable, as seen in enhanced *Interleukin-1β* (*IL-1β*) and *Interleukin-8* (*IL-8*) levels (Fig. S1). In contrast to that, no increase of *Interleukin-6* (*IL-6)* was detectable after 24 h of co-cultivation (Fig. S1B). Analysis on protein level did show enhanced IL-6 expression (Fig. S1D). Furthermore, expression of pleiotropic cytokines Interleukin 12 (IL-12) and 18 (IL-18) was slightly downregulated, in contrast to inflammatory tumor necrosis factor α (TNF-α) and angiogenesis/inflammation placenta growth factor (PIGF-2) expression (Fig. S1D). Interestingly, interferon β (IFN-β) levels were already slightly upregulated (Fig. S1D).

**Figure 1:**
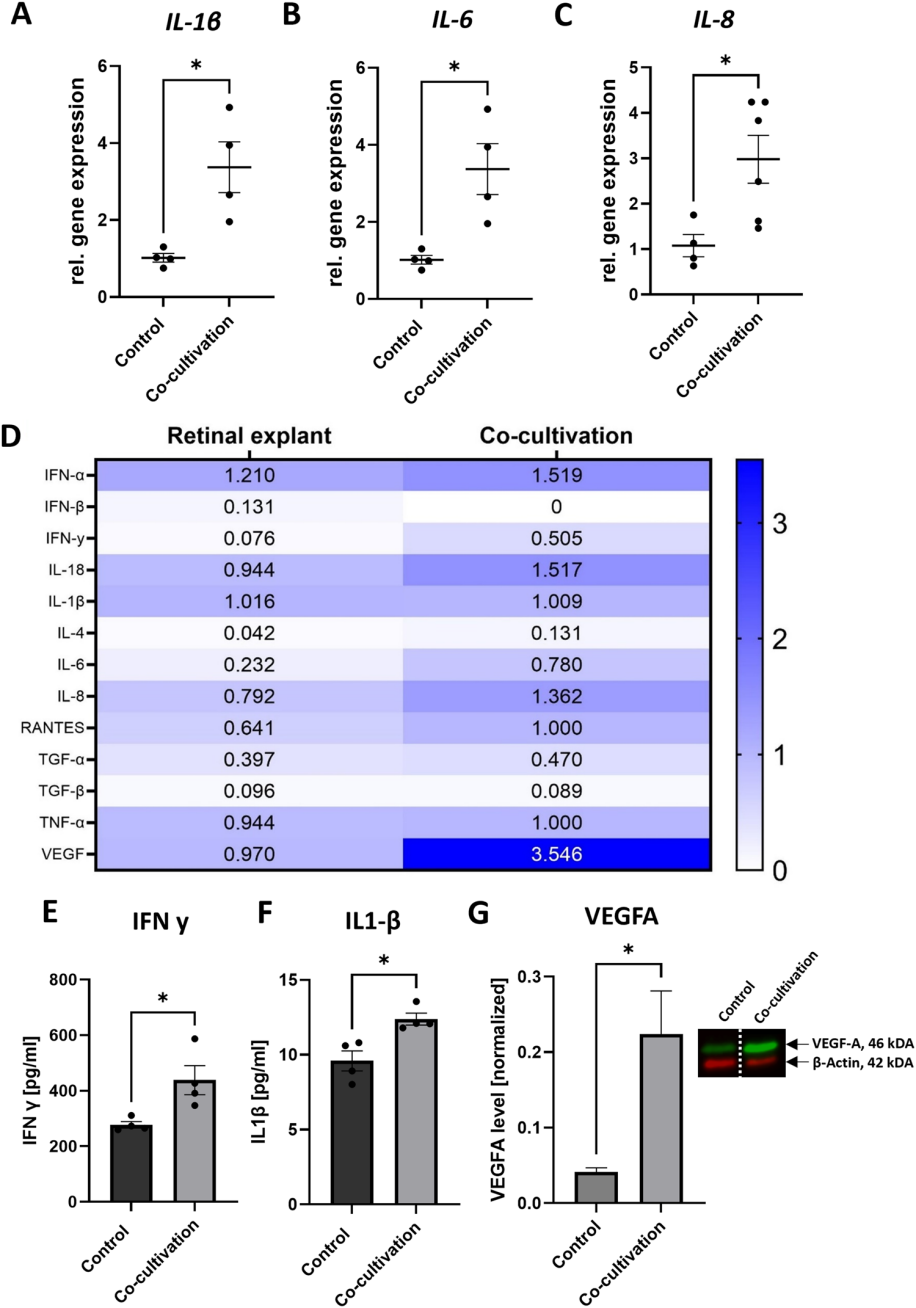
Direct co-cultivation over 48 h led to enhanced expression of pro-inflammatory cytokines. Porcine retinal explants were co-cultivated with primary, porcine RPE monolayers for 48 h. (**A–C**) Gene expression of pro-inflammatory cytokines *IL-1β,* *IL-6* and *IL-8* were significantly upregulated compared to control retinal explants. (**D**) Supernatants of five retinal explants or co-cultivated retinal explants were collected, and a semi-quantitative cytokine array was performed. Protein expressions of pro-inflammatory cytokines (IFN-γ, IL-1β, IL-4, IL-6) and VEGFA were upregulated. (**E,F**) IFN-γ and IL-1β levels were further evaluated using ELISA. Media of four retinal explants or co-cultivated retinal explants with RPE monolayers was collected and used in duplicates. (**E**) IFN-γ expression as well as (**F**) IL-1β levels were significantly enhanced in the media of co-cultivated retinal explants. (**G**) VEGFA protein abundance quantified by western blot demonstrated a significant increase due to co-cultivation. N = 4. *h* hours*,* *control* retinal explants without co-cultivation. Full blot in Fig. S2. Mean ± SEM is shown. Students-t-test. *p < 0.05*.*

After 48 h the expression of pro-inflammatory cytokines was strongly and significantly enhanced (*IL-1β* + 3.3-fold ± 0.67, p < 0.5; *IL-6* + 28.2-fold ± 6.08, p < 0.05*;* *IL-8* + 2.8-fold ± 6.08, p < 0.05), compared to control retinal explants (Fig. 1A–C). To verify if the expression of these cytokines is also enhanced on the protein level, a semi-quantitative cytokine array was performed. Besides a strong increase in IL-6 (+ 234%), Interleukin-4 (IL-4)(+ 209%), IL-18 (+ 59%) and IL-8 (+ 72%), interferon-γ (IFN-γ)(+ 614%) expression was also strongly increased in the supernatant of co-cultivated retinal explants (Fig. 1D). Interestingly, protein expression of angiogenesis marker VEGF was also strongly induced (+ 260%) due to the co-cultivation (Fig. 1D), nevertheless there was no increase in the gene expression in the co-cultivated retinal explant (Data not shown). The expression of RANTES (regulated and normal T cell expressed and secreted), a known inducer of inflammatory cascades upregulated in many inflammatory diseases like AMD^35,36^, was upregulated due to co-cultivation (+ 56%) (Fig. 1E). To further quantify the protein expression of IL-1β and IFN-γ, an ELISA with the supernatant of co-cultivated and control retinal explants was performed. Interestingly, IFN-γ levels, indicating the activation of resting microglia, was significantly induced on supernatants of co-cultivated retinal explants (+ 1.58-fold ± 53, p < 0.05) (Fig. 1E). Also, IL-1β expression was induced by 1.29-fold ± 0.78 (p < 0.5), marking an induced inflammation (Fig. 1F). The induction on protein level of VEGF (Fig. 1G, Fig. S2) was shown by quantification with western blot.

**Figure 2:**
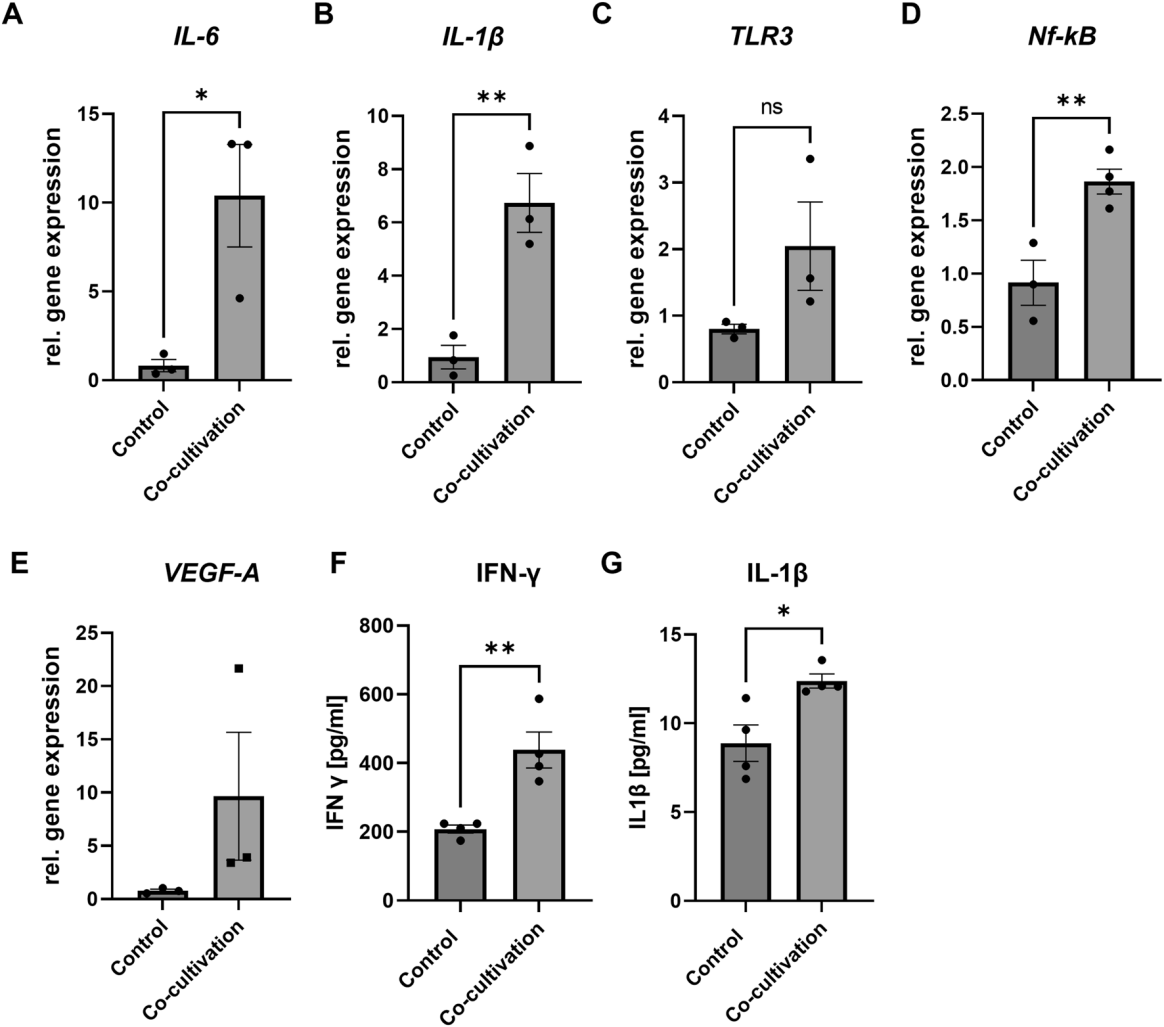
Co-cultivation led to increased pro-inflammatory gene expression in RPE-monolayers. Porcine retinal explants were co-cultivated with primary, porcine RPE-monolayers for 48 h. (**A,B**) Gene expression of pro-inflammatory cytokines *IL-6* and *IL-1β* was significantly upregulated in co-cultivated RPE-monolayers, compared to control RPE-monolayers. (**C**) Due to co-cultivation *toll-like-receptor* *(TLR*)-3 expression was upregulated in RPE-monolayers. (**D**) Multiplayer *Nf-kB* was significantly upregulated due to co-cultivation, probably furthermore enhancing inflammation. (**E**) Angiogenesis marker *VEGFA* was strongly, but not significantly, upregulated in the co-cultivated RPE-monolayer. (**F,G**) A significant increase in pro-inflammatory cytokine expression (IFN-γ, IL-1β) in the supernatant of (four) co-cultivated RPE-monolayers was demonstrated via ELISA. *h* hours*,* *control* RPE-P1-monolayers. (Welch’s-) t-test. *p < 0.05; **p < 0.01.

To further investigate the effects on the RPE monolayer, gene expression of pro-inflammatory cytokines in RPE monolayers were analysed (Fig. 2). Direct co-cultivation led again to an increase in *IL-6* and *lL-1β* expression (+ 12.6-fold ± 2,9, p < 0.05; + 7.11-fold ± 1.18, p < 0.01) (Fig. 2A, B). This demonstrates that inflammatory processes are also induced in the RPE-monolayer. Interestingly, *toll-like* *receptor* *3* *(TLR-3)* expression showed a slight tendency, although not statistically significant, to be increased due to co-cultivation (Fig. 2C), likely based on the activation of the Damage and Pathogen Associated Molecular Patterns (DAMP)-mediated TLR-3 pathway in the RPE cells. Multiplayer *nuclear* *factor* *kappa* *B* (*Nf-kB*) was furthermore significantly (p < 0.01) upregulated due to co-cultivation and could thus enhance inflammatory processes (Fig. 2D). VEGFA expression was strongly, but not significantly, increased in RPE-monolayers due to co-cultivation (Fig. 2E). As seen before in the retinal explants, secretion of IFN-γ (+ 2.1-fold ± 53.5, p < 0.01) as well as IL-1β (+ 1.4-fold ± 1.02, p < 0.5) was significantly upregulated in the supernatant of co-cultivated RPE monolayer (Fig. 2F, G).

An enhanced VEGFA protein expression, as seen in the supernatant of co-cultivated RPE-monolayers/retinal explants (Fig. 1D, G), was also detectable in RPE-monolayers (Figure S3). As RPE-cells are known to secrete VEGFA under normal culture conditions^37^, a detection of a slight expression in the not co-cultivated RPE-monolayer was not surprising. Co-cultivation strongly increased these levels in RPE-monolayers (Fig. S3).

### Co-cultivation induced inflammation causes autophagy dysfunction and neutral lipid deposits in RPE-monolayer

**Figure 3:**
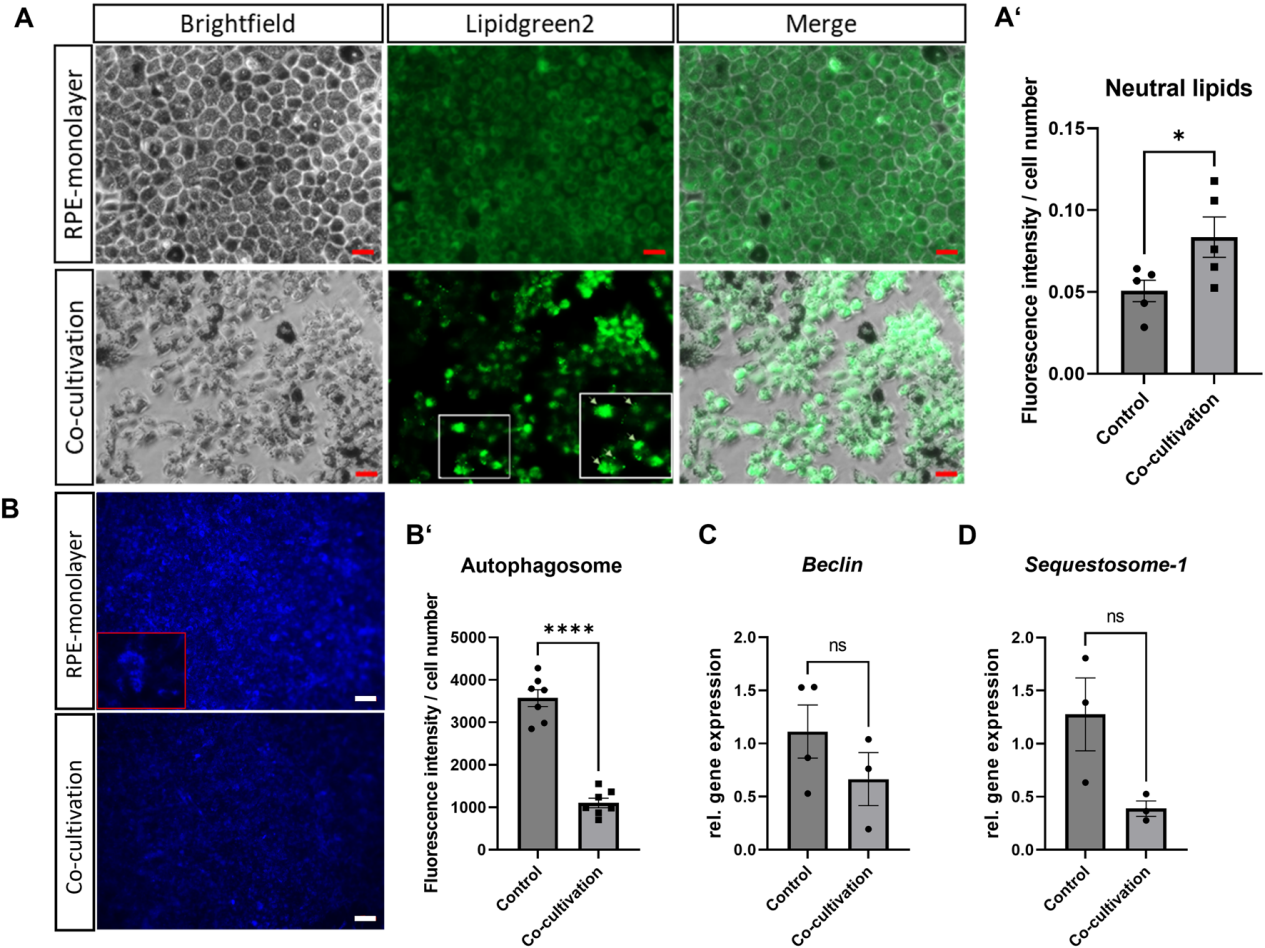
Direct co-cultivation led to neutral lipid deposits in the RPE-monolayer. Primary porcine RPE-monolayer were directly co-cultivated with retinal explants for 48 h. (**A**,**A’**) Without co-cultivation, neutral lipids (Lipidgreen2) were located homogenously in the cytoplasm of the RPE cells. Due to co-cultivation, neutral lipids accumulated and showed significantly increased fluorescence signals. Representative pictures are shown. Red scale bar 50 µm. N = 5 (for each 4 images were taken and analysed. The mean fluorescence was determined after background correction using ImageJ and normalized to cell number. (**B,B’**) Less autophagosomes were found in co-cultivated RPE-monolayers, underlining the autophagy dysfunction. The mean fluorescence was determined using ImageJ after background-correction and further normalized to cell number. N = 7 (for each 4 images were taken and analysed). White scale bar 100 µM. (**C**) The downregulation of *Beclin-1* stands for a dysfunction of autophagy in the RPE cells, probably caused by the inflammation. (**D**) The mRNA expression of autophagy-marker *Sequestomsome-1* was downregulated in co-cultivated RPE monolayers. *Control* RPE-P1-monolayers. Mean ± SEM is shown. (Welch’s-) t-test. ****p < 0.0001.

Dysfunctional RPE cells accumulate lipid deposits, called drusen^38^. Besides esterified cholesterol and apolipoproteins, neutral lipids are abundant in the deposits and drusen of AMD eyes^39^. Lipidgreen2 staining was used to stain neutral lipids and revealed an increased and accumulated existence for lipids in the destroyed RPE monolayer (Fig. 3A, A’). In general, neutral lipids were found homogenously distributed in the cytoplasm of not co-cultivated RPE cells, in contrast to a dot-like pattern observed in co-cultivated RPE cells (Fig. 3A). Therefore, the co-cultivation resulted in accumulated neutral lipid deposits. In this context, also a strong cell loss was visible after 48 h of co-cultivation (Fig. 3A, Brightfield image).

One cause of accumulated proteins in cells is a disturbed autophagy, which is furthermore known to be increased due to apoptosis, as caspase activation reduces the autophagic process^40^, and plays a role in AMD pathogenesis^41,42^. Therefore, autophagosome staining by a fluorescent autophagosome marker was performed to investigate the intracellular degradation system after 48 h of co-cultivation (Fig. 3B). The mean fluorescence signal, representing the amount of stained autophagosomes, was significantly decreased in co-cultivated RPE-monolayers (−56%, p < 0.0001) (Fig. 3B’). Therefore, autophagy was impaired in the co-cultivated RPE cells. This disturbance was also seen in a decrease of *Beclin-1* expression, compared to control RPE-monolayers (Fig. 3C), as well as a reduction in *Sequestosome-1* expression (Fig. 3D). Taken together, the seen accumulation of neutral lipids due to inflammation, was probably caused by impaired autophagy of the damaged RPE-cells.

### Inflammation caused by co-cultivation resulted in cell death in retinal explant as well as in the RPE-monolayer

**Figure 4:**
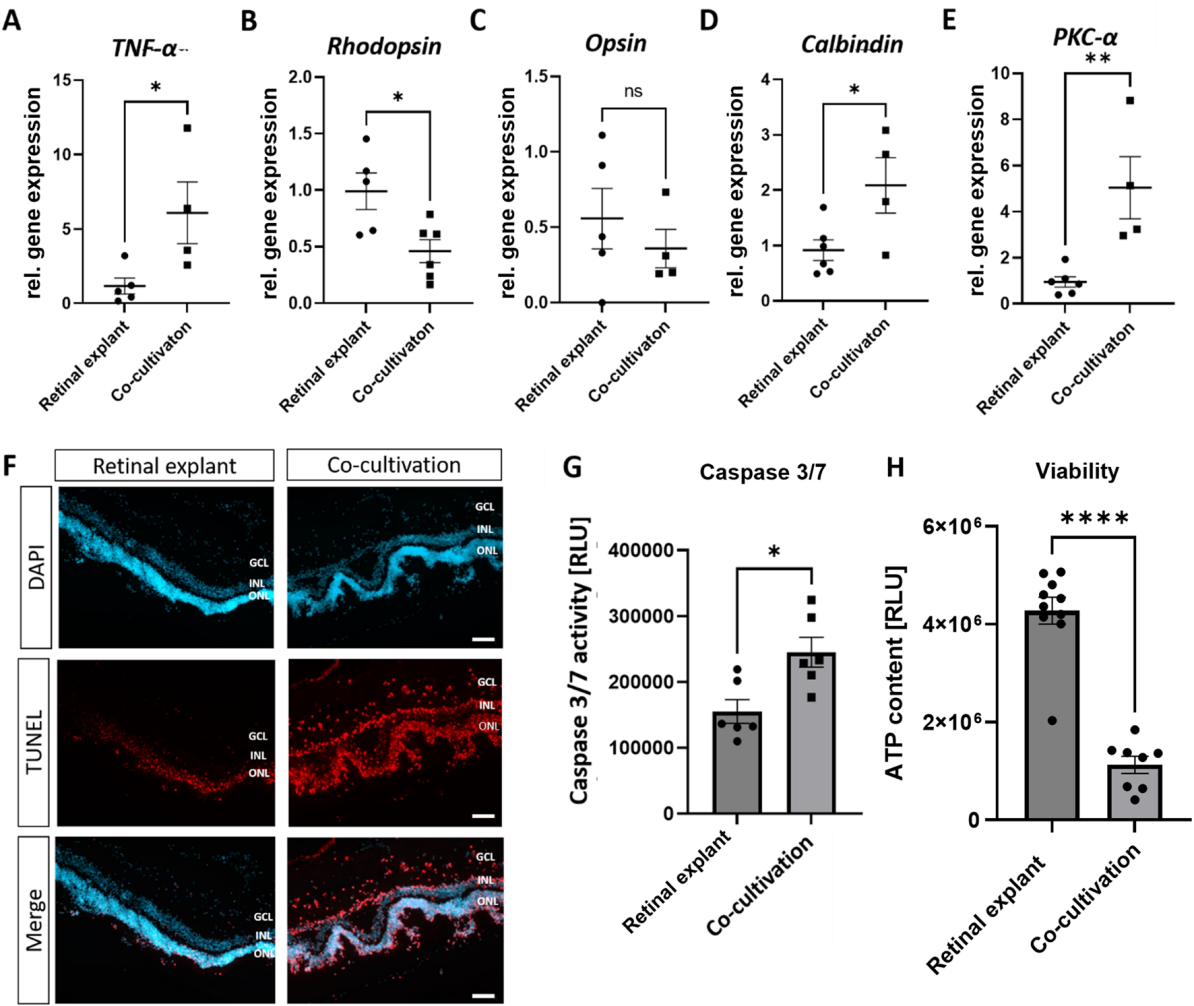
Direct co-cultivation led to cell death in the retinal explant. Retinal explants were directly co-cultivated with a primary porcine RPE-monolayer for 48 h. (**A**) Gene expression of cell death marker *TNF-α* was significantly upregulated due to co-cultivation. (**B,C**) Photoreceptor markers *rhodopsin* and *opsin* were downregulated in co-cultivated retinal explants. (**D,E**) Co-cultivation led to increased *calbindin* and *PKC-α* expression. (**F**) More TUNEL+, thus apoptotic, cells were found in all retinal layers of the co-cultivated retinal explant. Representative pictures are shown. N = 3. (**G**) Apoptosis induction was further confirmed with higher caspase 3/7 activity of the co-cultivated retinal explant. Experiments were repeated three times with similar results. (**H**) Direct co-cultivation led to a significant loss in the viability of the retinal explant, as seen in a strong reduction of ATP levels. Experiment was repeated three times with similar results. *GCL* Ganglion cell layer, *INL* inner nuclear layer, *ONL* outer nuclear layer. White scale bar 100 µM. Mean ± SEM is shown. (**G,H**) Experiments were repeated three times with similar results. (Welch’s-) t-test. *p < 0.05; **p < 0.01; ***p < 0.001; ****p < 0.0001.

In co-cultivated retinal explants, *TNF-α* expression was increased (+ 5.5-fold ± 1.9, p < 0.05) (Fig. 4A), which could also indicate a pro-apoptotic function, in addition to its pro-inflammatory role. Furthermore, expression of rod-photoreceptor marker *Rhodopsin* was significantly reduced (−46%, p < 0.05), likely due to the cell death of this retinal cell type (Fig. 4B). *Opsin* expression, a marker for cone-photoreceptors, was not significantly altered (Fig. 4C). In contrast to that, *calbindin* (horizontal cells) and *protein* *kinase* *C-alpha* (*PKC-α*,retinal bipolar cells) expression was significantly (p < 0.05; p < 0.01) increased in co-cultivated retinal explants.

To further verify the observed cell death due to co-cultivation, TUNEL-staining against apoptotic cells was performed. A strong increase of TUNEL+ cells was observed in all retinal layers after 48 h of co-cultivation (Fig. 4F). Therefore, co-cultivation may induce apoptosis in retinal explants, which was further quantified and analysed with caspase 3/7-activity measurements (Fig. 4G). A significant increase of caspase 3/7-activity was detected in co-cultivated retinal explants (+ 25%, p < 0.05) (Fig. 4B), together with a significant loss of adenosine triphosphate (ATP)-levels (-74%, p < 0.0001) (Fig. 4D). A strong loss of ATP-levels likely indicates mitochondrial cell death, e.g., secondary necrosis^43–45^.

**Figure 5:**
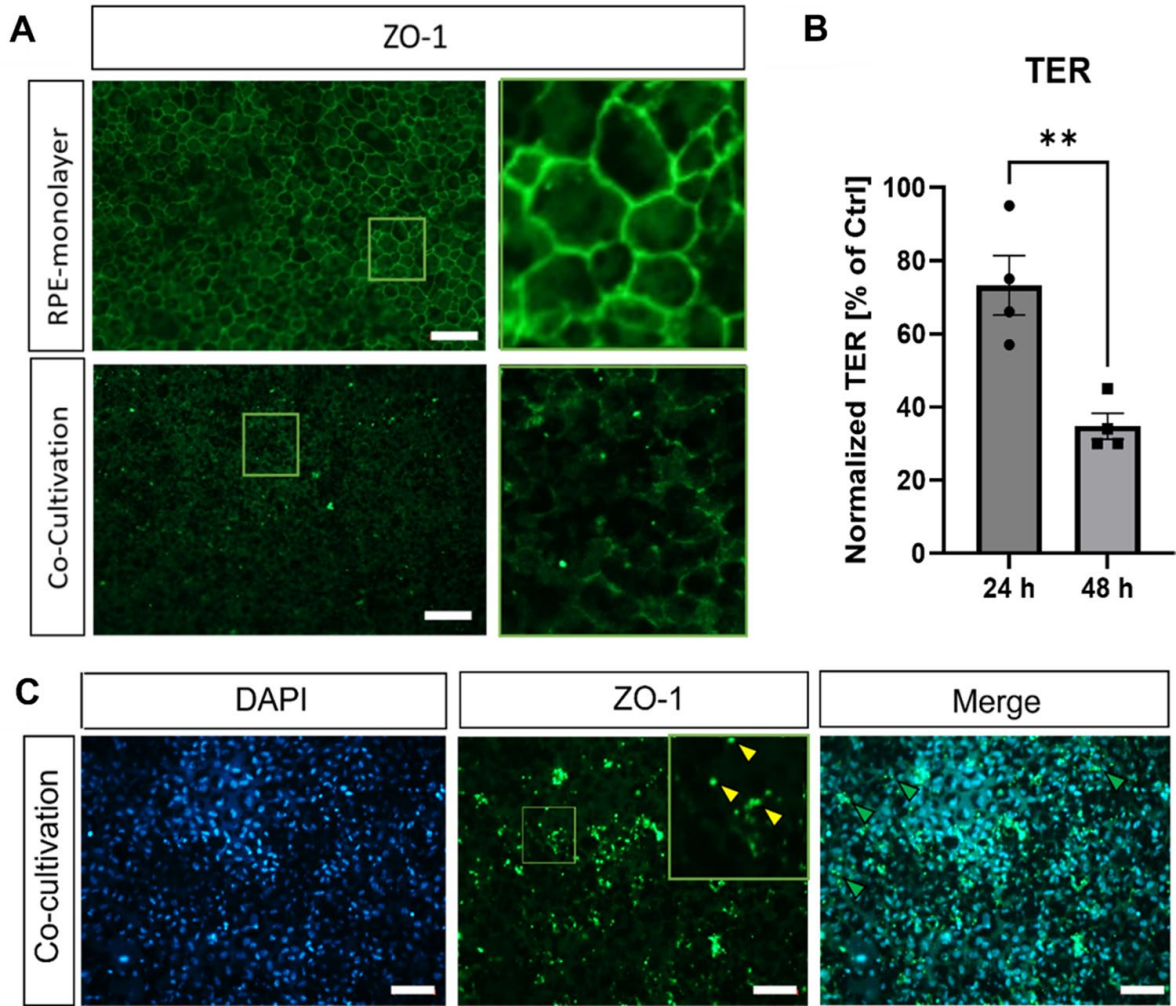
Tight-junction marker ZO-1 loss in RPE-monolayers was caused by co-cultivation. Primary RPE-monolayers were co-cultivated for 48 h. (**A**) Tight-junction marker ZO-1 was strongly reduced in co-cultivated RPE-monolayers. (**B**) TER-values of RPE-monolayers were measured before and after 24 and 48 h of direct co-cultivation. Data was expressed as the fold-changes in percentage from the starting TER value (not yet co-cultivated). The TER of direct co-cultivated retinal monolayers decreased significantly over time. N = 4. (**C**) In parts of the co-cultivated RPE-monolayer, ZO-1 expression accumulated (yellow arrows) mostly in between the RPE cells (green arrows). (**A,C**) Representative pictures are shown, n = 4. Scale bar: 50 µM. Mean ± SEM is shown. Students t-test. **p < 0.01.

Direct co-cultivation led to a loss of typical, membrane-bound ZO-1 staining after 48 h (Fig. 5A). TER measurements demonstrated a significant decrease over time (−62% after 48 h, p < 0.01) (Fig. 5B), underlining the loss of the tight monolayer. Furthermore, in parts of the RPE-monolayer, probably in direct contact with the retinal explant, ZO-1 accumulated (yellow arrows) between the RPE cells (green arrows) (Fig. 5C).

Direct co-cultivation resulted further in a disruption of the RPE monolayer over time (Fig. S4B–D), together with increased number of clumps of dead RPE cells, and an accumulation of pigment (Fig. S4B–D). After 48 h of co-cultivation, first indicators of these effects were detectable (Figure S4B), increased over 4 days (Fig. S4C) and ended with cell death of almost all RPE cells after 7 days (Fig. S4D).

**Figure 6:**
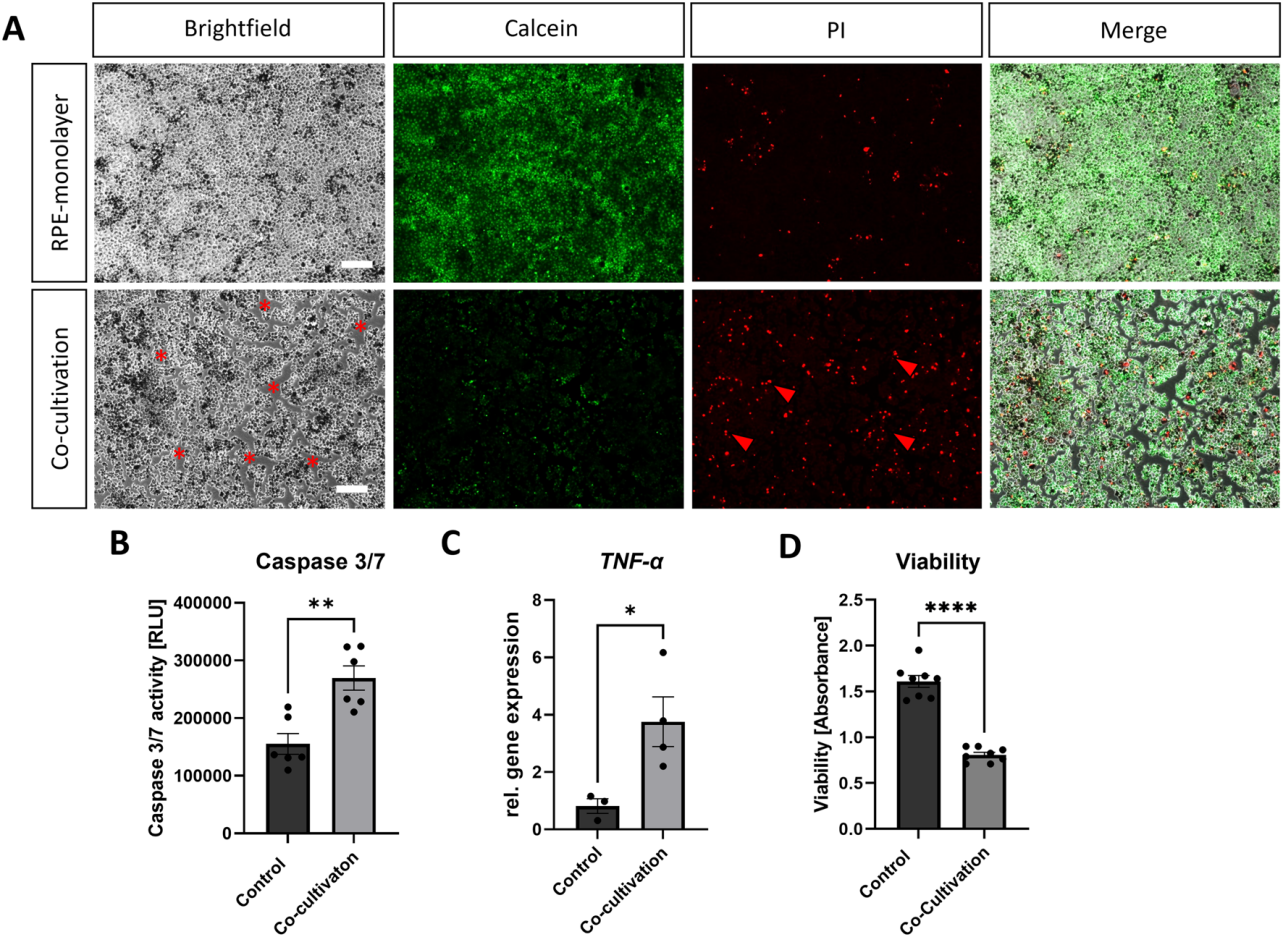
Co-cultivation led to cell-death in the RPE monolayers. Primary RPE-monolayers were co-cultivated with porcine retinal explants for 48 h. (**A**) More dead (PI+, red arrows) and less alive (Calcein+) RPE cells were detected in the co-cultivated RPE-monolayer. Red stars mark disrupted areas of the co-cultivated RPE-monolayer. Representative pictures are shown, n = 4. (**B**) Induction of cell death was quantified by caspase 3/7 activity. Co-cultivation led to a significant increase of caspase 3/7 activity in RPE-monolayers and thus apoptosis in these cells. Experiment was repeated three times with similar results. (**C**) Gene expression of apoptosis-marker *TNF-α* was significantly enhanced in co-cultivated RPE-monolayers. (**D**) Co-cultivation resulted in a significant loss of viability in the RPE cells. Experiments were repeated three times with similar results. Scale bar: 200 µM. Mean ± SEM is shown. Welch’s t-test. *p < 0.05; **p < 0.01; ***p < 0.001; ****p < 0.0001.

This disruption is probably caused by the cell death of the RPE cells, as Live/Dead staining revealed more dead [Propidium iodide (PI)+, red arrows] and less alive (Calcein+) cells in the co-cultivated RPE-monolayer after 48 h (Fig. 6A). Also, a disruption of the RPE-monolayer was visible in phase-contrast microscopy (Figure S4, white stars) (Fig. 6A, red stars). Cell death was further quantified by caspase 3/7 activity, which was significantly enhanced (+ 1.74-fold, p < 0.01) (Fig. 6B) in co-cultivated RPE-monolayer, resembling caspase-mediated apoptosis. Likewise, an increased expression *TNF-α* (+ 4.64-fold ± 1.04, p < 0.05) was detectable in co-cultivated RPE-monolayers (Fig. 6C). It has been described that TNF-α induced inflammatory gene signalling can switch to cell death via apoptosis or necroptosis, thus enhanced TNF-α expression could increase cell death of the RPE cells^33^. Furthermore, cell viability was significantly decreased (−46%, p < 0.0001) (Fig. 6D). Therefore, the inflammation caused by direct co-cultivation resulted in cell death in the RPE monolayer and thus to a disruption of the RPE-monolayer and deposit of neutral lipids.

Interestingly, this effect seems to depend strongly on the primary properties of the RPE cells in the co-cultured monolayer. Thus, co-cultured RPE-P1 monolayers differ markedly from RPE-P6 monolayers in the cytokines they secrete (Fig. S4A). Co-cultivation with a P6 monolayer results in noticeably reduced expression of, among others, pro-inflammatory cytokines (Fig. S4A). This, in turn, seems to have an influence on the degeneration induced by co-cultivation, e.g., caspase 3/7 activity is not increased with P6 monolayers (Fig. S4B). Furthermore, only co-cultivation with a primary P1 monolayer led to a reduction of the ATP content of the retinal explants, but not when the explants are co-cultured with P3 or P6 monolayers (Fig. S4C). Similarly, IFN-γ expression is not increased when the explants are co-cultured with P3 monolayers. Accordingly, RPE monolayers can be co-cultured with retinal explants without inducing inflammation and degeneration when they have partially or completely lost their primary character.

## Discussion

Organ retinal cultures provide an important link between 2D-cell cultures and in vivo-models, taking advantage from maintaining heterogeneous cell populations that can be observed in situ. Only few publications have included primary RPEs in their ex vivo retinal models, and those that did only used a non-confluent RPE culture in an indirect manner. This study aimed to directly co-cultivate a primary, porcine RPE-monolayer with porcine retinal explants to evaluate the potential of this model to simulate inflammatory diseases or immunologic rejections.

**Figure 7:**
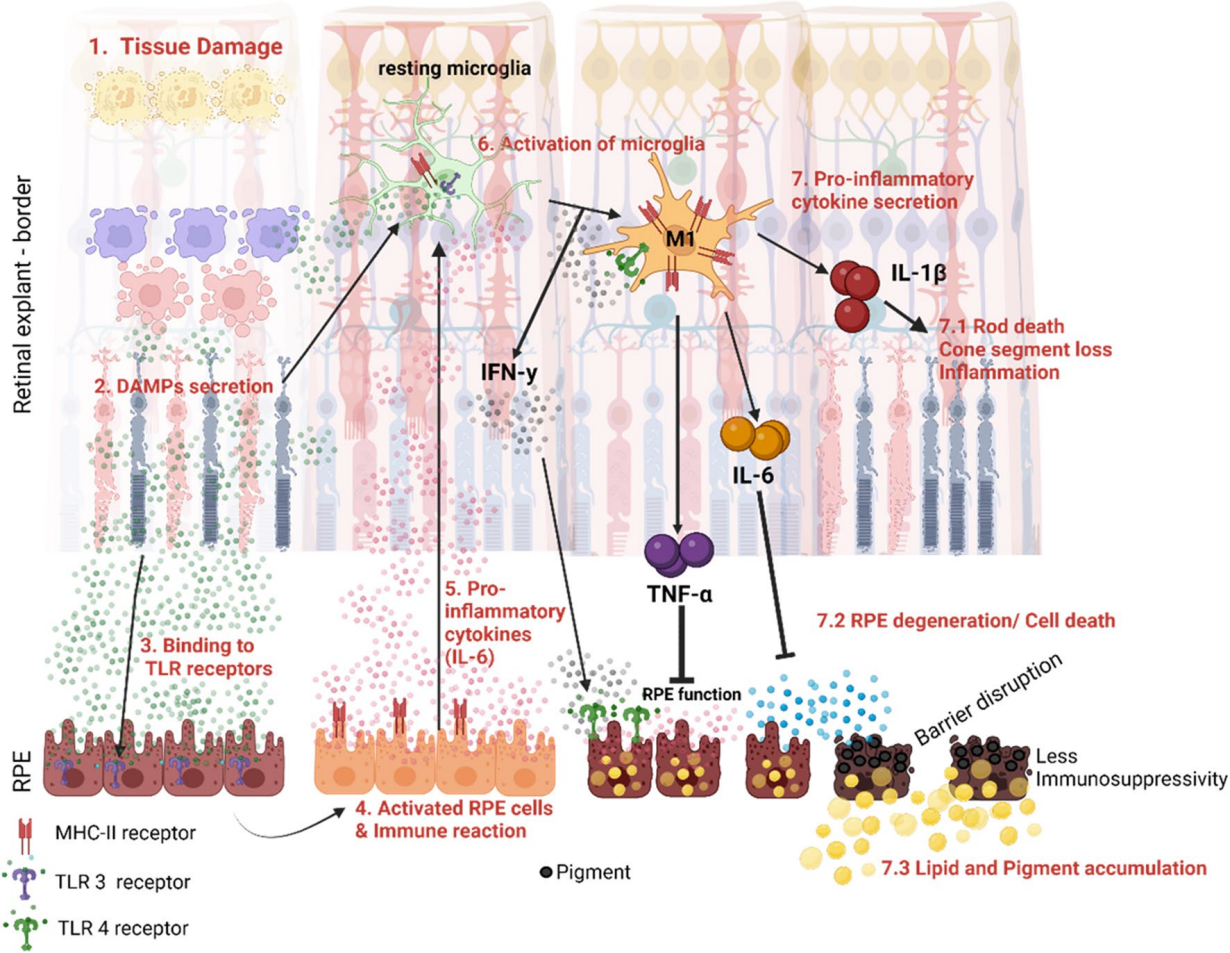
Tissue damage results in activation of RPE cells, inflammation, and cell death in a direct co-cultivation model. The generation of retinal explants causes (**1**) tissue damage on the edge of the circular explant. (**2**) Due to the tissue damage, DAMPs (danger associated molecular pattern) are secreted, e.g., RNA from the dying cells. (**3**) DAMPs are known to bind to toll-like-receptor (TLR)-3 in retinal pigment epithelium (RPE) cells. (**4**) The binding to the TLR-3 receptor results in the activation of the RPE cells and thus in an immune response. (**5**) RPE cells therefore secrete pro-inflammatory cytokines, e.g.*,* IL-6. (**6**) IL-6, as well as DAMPs, can be recognized from resting microglia in the tissue and transform them into activated microglia. This transformation causes the secretion of IFN-γ, which can be recognized by TLR-4 of RPE cells. (**7**) The activation of microglia results in the secretion of various cytokines*,* e.g., IL-1β, IL-6 or TNF-α. This results in (**7.1**)**.** Photoreceptor cell death and inflammation as well as to (**7.2**) degeneration and cell death of RPE cells, resulting in the disruption of the barrier function and less immunosuppressive functions. (**7.3**) Furthermore, pigment is accumulated in the cells as wells as neutral lipids, maybe resembling initial stages of drusen.

Inflammation resembles a cellular reaction to foreign or damaged material and aims to eliminate these factors. Damaged or foreign material is recognized by different pattern recognition receptors, resulting in the activation of intracellular pathways and the production of various pro-inflammatory cytokines. Damaged cells secrete molecules named damage-associated molecular patterns (DAMPs), which can be recognized by a variety of cells expressing the corresponding receptors, inducing an immune response. Thus, tissue injury, likely happening due to punching the retina to generate retinal explants, could initiate an inflammatory response through DAMPs (Fig. 7).

RPE cells form the first line of defence against danger signals by expressing almost all TLR isoforms, with TLR-3 being the most expressed^46,47^. Klettner et al*.* demonstrated that the activation of TLR2, -3, and -4 induces pro-inflammatory response in the RPE cells that could play a part in long-term inflammation, reduce RPE function, and result in RPE degeneration^48^. Therefore, the activation of TLRs in RPE could intensify the inflammatory response, which contribute to AMD development. As TLR-3 receptors are activated by DAMPs from dying cells, and TLR-3 activated RPE cells have been shown to exacerbate inflammatory response of microglia, our results suggest that RPE cells, when functional in monolayers, can react to tissue damage and induce an inflammatory response (Figs. 2, 7). This would also explain why increased IL-6 protein levels were found in the supernatant of co-cultivated retinal explants, but no induced gene expression in the retinal explants, as RPE cells secrete IL-6 after their activation via TLR-3 (Fig. 1). Besides a upregulated caspase 3/7 activity (Fig. 6B), *TLR3*-expression was upregulated in RPE cells (Fig. 2C) which induces, besides its pro-inflammatory effect, cell death in RPE cells^49,50^. The increased amount of PI + RPE cells (Fig. 6A) suggests either apoptosis or necrosis as cell death pathways, as PI can only penetrate cells which lost their membrane integrity. During necrosis the cell membrane loses its selective permeability, thus PI can enter the cells. In contrast, during initial apoptosis, the cell membrane remains intact. However, in late apoptosis, necrosis-like membrane disintegration happens, thus PI also enters the cells. Therefore, PI staining cannot differentiate between necrotic cells and late apoptotic (secondary necrosis) cells. It is important to note, that insufficient clearance of apoptotic cells can also result in secondary necrosis, thus also resulting in the release of DAMPs and enhancing inflammation^29,51^. As there is no clearance of dead RPE cells, secondary necrosis / late apoptosis is likely to happen. Because caspase 3/7 activity was induced due to co-cultivation, RPE cells may die primarily due to apoptosis (Fig. 6B). The decision, which pathway is activated, may be dependent on the amount of activated TLR-3^48^. The inflammation in our co-cultivation model led also to increased caspase 3/7 activity in the retinal explant (Fig. 4G), which is also observed as apoptosis in the pathogenesis of AMD^52^.

Enhanced IL-1β levels resulted in human ARPE-19 cells in a decrease of TER values and permeability, standing for altered tight junctions^53^. Therefore, either IL-1β by itself or its downstream targets could enhance the dysfunction and disruption of the co-cultivated RPE monolayer (Fig. S4, Fig. 5). Compared to the supernatant of control RPE-monolayers, co-cultivation led to a significant increase in IL-1β levels (Fig. 2G). This indicates that the increased IL-1β levels were secreted from the RPE-monolayers. Therefore, activated RPE cells are probably the main inflammatory trigger in this damage co-cultivation model. RPE cell death (Fig. S4; Fig. 6), is perhaps the primary reason that the RPE barrier was disrupted and resulted in decreased TER values (Fig. 5B). Viable, primary RPE cells in monolayers normally demonstrate a TER value of 200–400 Ω cm^2^^54^, similar values to non-cultivated RPE monolayers in this study (~ 300 Ω cm^2^).

VEGFA, a marker for angiogenesis, is secreted from RPE cells under normal conditions^55^ and can be induced by inflammation^56^. In addition, VEGF secretion can also be caused by TLR-3 activation^56,57^. As mRNA levels only increased in co-cultivated RPE-monolayers (Fig. 2E), as well as protein expression (Fig. S3), the main source of VEGFA is probably the activated RPE-monolayer, thus inducing angiogenesis in the inflamed retinal explant. Excessive levels of VEGF promote neovascularization and thereby contribute to the development of wet AMD^58^. VEGF, and other cytokines have been furthermore implicated in the disruption of RPE barrier functions and lead in ARPE-19, as well as in porcine RPE cells, to a significant drop in TER^59^.

Recent studies of atrophic AMD suggest that inflammation initiated by RPE cell inflammatory responses and cell death play an important role in drusen biogenesis^60^. Drusen represent immunologically active deposits, containing mostly lipids, and may function as additional trigger for immune responses in the eye. In primary porcine RPE cultures, cultivated on trans-well inserts for over six weeks, deposits were already detected^61^. Likewise, in our RPE-monolayers, after four weeks of cultivation, neutral lipids were found in a uniform manner (Fig. 3A). These neutral lipids accumulated due to co-cultivation (Fig. 3A), probably due to the inflammation caused dysfunction of the cells. Therefore, the observed accumulation in co-cultivated RPE-monolayers could stand for a primitive and early deposit, similar as seen in the beginning of inflammatory diseases like AMD. The dysfunction of the RPE cells was also detectable in a loss of its autophagy functions (Fig. 3B–D), probably resulting in the accumulation of neutral lipids (Fig. 3A). It is known, that insufficient digestion, due to impaired autophagy of the RPE cells, lead to the accumulation of damaged and/or toxic proteins, as well as lipofuscin, and extracellular drusen deposits^41^. All of them further contributes to RPE dysfunction and death and have been associated with the pathogenesis of AMD^42^.

In chronic retinal disorders such as AMD, dysregulated inflammation is a main cause. As a result, increased pro-inflammatory cytokines, such as TNF-α, IL-1β, IL-6 and IL-8 contribute to further progression of the disease, as seen in our co-cultivation model (Fig. 1). These secondary mediators of inflammation are released by activated immune retinal or RPE cells, thus can be detected in the co-cultivated retinal explant as well as in the RPE-monolayer (Figs. 1, 2). Therefore, the retinal explant as well as the RPE cells may trigger each other in these inflammatory processes and thus enhance and fasten inflammation. This would also explain why cell death occurs after such a short co-cultivation time. At this point, it is important to emphasize that the progressive aspect of neurodegenerative diseases can hardly be mimicked with ex vivo models, which is based on the relatively short cultivation period of the organ cultures. In relation to the possible "lifetime" of a retinal explant, 48 h of co-cultivation is already 1/4 of the cultivation time. Our data also suggest that the strong inflammation depends on the primary character of the RPE cells (Fig. S5). Co-cultivation with higher passages reduced pro-inflammatory expression and degeneration (Fig. S5). Therefore, if wanted, co-culturing with higher passages may be used to create a non-inflammatory model. However, this would first require clarification of the extent to which these monolayers are still functional and indeed still have supportive properties. It is also important to note, that the foreign origin of RPE cells, as they come from various animals and have thus a high genetic variance, could induce an immune response in the co-cultivated retinal explant. In transplanted allogenic human RPE cells a rejection has been shown in vivo^62^.

Also, as co-cultivation with human ARPE-19 cells showed to have partly positive effects on the co-cultivated retinal explant^63^, it may be of interest to investigate this co-cultivation further. Interestingly, in contrast to the recent co-cultivation data^63^, negative, inflammatory effects have been reported before, as seen in gliosis and complement system activation in the retina as well as in the ARPE-19 cells^64^. In both publications, sub-confluent ARPE-19 cells were used (5 weeks^64^ and 24 h^63^ old). In contrast to Wagner et al., Mohlin et al. used a direct co-cultivation, probably resulting to enhanced inflammation due to close contact. Data on TLR activation in ARPE-19 cells are controversial, with TLR3 and 4 being expressed^65^ but studies demonstrating both activation by LPS^66^ and lack of activation^67^. Therefore, it cannot be excluded that this cell line does not react like primary RPE cells or can only react in an attenuated manner. RPE-monolayer generated by iPSC-RPE derived from donors with AMD demonstrated decreased mitochondrial function and an elevated inflammatory marker, compared to iPSC-RPE from donors without AMD^68^. Furthermore, co-cultivation of Factor H (FH) deprived hTERT (immortalized human RPE) cells with porcine retinal explants resulted in changes including the mitochondria and lipid composition, as well as retinal degeneration, which was independent of glial cell activation. As one leading genetic risk associated with AMD is FH substitution, this study demonstrates that active, pro-inflammatory RPE cells can damage the retina. Neurodegeneration was also induced in our co-culture, which was furthermore also independent of (Müller) glia cells (data not shown). However, to the best of our knowledge, no data is available concerning the TLR-expression and pathway activation in hTERT-RPE1 cells. In summary, immortalized cell lines, like the ARPE-19 or hTERT cells, often lack some physiological characteristics of RPE in vivo, so that their use always comes with a potential trade-off compared to primary cells.

It is known that during retinal degeneration, activation of Müller cells can play a role as well as activated microglia. The activation of Müller cells can be measured by GFAP expression, a marker of gliosis, which is upregulated due to retinal stress and results in degenerative changes of the inner retina and neurodegeneration in retinal diseases such as AMD. It was also demonstrated before, that there is an interplay between activated Müller cells and microglia^69^. Although we have previously shown that 24 h of co-cultivation do not result increased *GFAP* gene expression^29^, it cannot be excluded that a longer cultivation period (48 h) induces up-regulation or increases GFAP at the protein level. Therefore, it is possible that Müller cells also play a role in the inflammatory degeneration described here and this should be investigated in further experiments. The switch to active microglia can be seen in enhanced IFN-γ levels. In the supernatant of co-cultivated retinal explants, increased expression of IFN-γ was detectable, consequently standing for the transformation of inactive microglia to pro-inflammatory microglia (Figs. 1D, E, 2F). An increase of IFN-γ levels was also observed in patients with AMD^70^. The stimulation of the TLR-3 receptor resulted in RPE cells in an enhanced production of IFN-γ, besides the secretion of also pro-inflammatory cytokines IL-6, IL-1β, IL-8 and TNF-α^71^. Thus, the enhanced IFN-γ levels in the co-cultivation could be produced partly also by RPE cells and may result in cell death of the RPE-monolayer, as seen in enhanced PI + cells (Fig. 6A) and induced caspase 3/7 activity (Fig. 6B). Interestingly, a recent study investigating identified IL-6 as a potential AMD target^72^, thus new therapy approaches could be tested in this damage model.

Dysregulated calcium (Ca^2+^)-homeostasis results also to neurodegeneration and is therefore observed in several neurodegenerative disorders, including AD^73^. Thus, excessive Ca^2+^ is thought to be a critical step in neurodegenerative progress and members of the Ca^2+^-binding protein family, e.g. calbindin, are thought to play a neuroprotective role in various pathologic conditions, by serving as a buffer against excessive Ca^2+^ calcium^74^. Concentrations of extracellular Ca^2+^ can increase due to inflammation^75^. The expression of calbindin is upregulated in this context, acting as a buffer against the rising intracellular Ca^2+^ levels^76^. Thus, the observed increased *calbindin* levels, besides representing a marker for horizontal cells, could also be a result of increased Ca^2+^ levels, caused by inflammation. This hypothesis is further underlined by the depletion of ATP in the retinal explant due to co-cultivation (Fig. 4H), as one cause can be Ca^2+^ overload^77^. Calbindin and PKC-α are known to co-localize and bipolar cells receive essential inputs from horizontal cells in the retina^78^. As co-cultivation also led to increased *PKC-α* levels (Fig. 4E), this may be a result of increased *calbindin* levels (Fig. 4D), as an increased crosstalk between these two retinal cell types is needed.

## Conclusion

Taken together, we demonstrate that direct co-cultivation with porcine RPE-monolayer and porcine retinal explant results in an inflammation-driven degeneration model, that can be used to investigate inflammatory neurodegenerative diseases like AMD. Another advantage of this model is the use age of porcine eyes, which require no breeding and killing of laboratory animals, as the eyes are by-products of the food industry.

## Material and methods

### Handling of pig eyes

Porcine eyes from euthanized 6-months-old pigs were obtained from a local abattoir. The eyes were instantly transported at 4 °C to the laboratory to ensure a maximum time of 3 h after death of the animals. Within 1 h after arrival of the eyes at the lab, retinal explants or primary cells were obtained. As a first step, eyes were cleaned from remaining tissue and the optic nerve was trimmed back without perforating the eyeball. Afterwards, the eyes were disinfected in 70% ethanol for five minutes. After washing in PBS, they were placed under a laminar flow hood. The remaining eyes were stored at 4–8 °C. One eye was placed in a petri dish lid, secured using forceps and a small line was cut with a sharp scalpel. Afterwards, a line was cut around the circumference of the globe (in 0.5 cm distance from the limbus) using sharp scissors. Cornea, lens, and vitreous were removed.

### Antibodies

Following antibodies were used in this study (Table 1):

**Table 1:** Antibodies and dilutions used in this study.

Antibody	Company	Species of origin	Type of antibody	Dilution
β-Actin	Cell signaling, Danvers, Massachusetts, USA	Rabbit	Primary, monoclonal	1:1000
VEGF	Santa-Cruz, #sc-7269, Dallas, TEXAS, USA	Mouse	Primary, monoclonal	1:100
VEGFA	Proteintech, Planegg-Martinsried, Germany	Mouse	Primary, monoclonal	1:200
ZO-1	Santa-Cruz, #sc-33725, Dallas, TEXAS, USA	Rat	Primary, monoclonal	1:100
Anti-rat	Li-Cor, #925-32219, Lincoln, NE, USA	Goat	Secondary. Polyclonal	1:1000
Anti-mouse	Li-Cor, #926-68070, Lincoln, NE, USA	Goat	Secondary, polyclonal	1:1000

### Isolation of RPE cells

Primary RPE cells were isolated from 16 porcine eye cups as described before^31,79^. Briefly, porcine eyes were opened in a cleave-like manner and the retina was removed completely. Afterwards, eyecups were digested for 15 min with Tryple-Express (Thermo Fisher Scientific, Karlsruhe, Germany) in the incubator and again for 45 min using Trypsin/EDTA (0,25%, Thermo Fisher Scientific, Karlsruhe, Germany). The digestion was stopped using 10% FBS/PBS (Thermo Fisher Scientific, Karlsruhe, Germany), cells were collected in two 50 ml falcon tubes and centrifuged at 800*g* for 7 min. After removing the supernatant, cell pellets were resuspended in 37 °C RPE-cultivation media (10% FBS, 1% Penstrep (PS), High glucose DMEM) (Thermo Fisher Scientific, Karlsruhe, Germany) and seeded in a 12-well plate. Cells were cultivated in an incubator (5% CO_2_ at 37 °C) and media was changed completely after 48 h. Then, 80% of the media was changed 3-times a week until cells reached confluence. From then onwards, 80% of the media was changed 3-times a week using RPE-confluence media (with only 1% FBS). If needed, cells were collected and seeded in a T25 flask containing 5 ml of RPE-cultivation media (until confluency was reached, then RPE-confluence media was used). Otherwise, cells were cultivated in 12-well plate to remain in a low passage number (P0). Only RPE cells in passage 1 or 2 were used to generate RPE-monolayers.

### Generation of RPE-monolayers

Primary RPE monolayers were generated as described previously^31^. Briefly, primary porcine RPE cells were isolated and cultivated for at least two weeks until reaching confluency (Paper). 12- or 24-well plate Transwell Inserts (ThinCerts, Greiner Bio-One, Frickenhausen, Germany) were coated with Laminin (20 µg/mL, Sigma-Aldrich/Merck, Darmstadt, Germany) the day before use and incubated overnight in the fridge. Shortly before seeding the cells, laminin was removed, and inserts were washed three times with 1xPBS. RPE cells (P0–P1) were counted and 300,000 cells for a 12-well plate insert were seeded in 10% FBS containing RPE cultivation media [4.5 g/L Glucose containing DMEM (Thermo Fisher Scientific, Karlsruhe, Germany) supplemented with 1% PS, 10% FBS (Thermo Fisher Scientific, Karlsruhe, Germany), 1% NEAA (Thermo Fisher Scientific, Karlsruhe, Germany), 0.25% HEPES Buffer (Lonza, Köln, Germany)]. In case of 24-well plate inserts, 150,000 cells were seeded on each trans-well insert in 10% FBS containing RPE cultivation media. After 48 h, cultivation media was refreshed completely. Next, three times a week the media was changed to 80%. When cells reached confluence, media was changed to 1% FBS containing RPE confluence media to further enhance correct polarization and to prevent overgrowing. After 4–5 weeks in culture, trans-electrical-resistance (TER) was determined and only inserts with values above 250 Ωcm^2^ and with a typical morphology (uniform pigmentation, honeycomb-shape) were used. Only primary RPE passages (P1-P2) were used for these experiments.

### Generation of porcine retinal explants and co-cultivation

Three hours before usage of the RPE-monolayers for co-cultivation, RPE confluence media was substituted to retina cultivation media [Neurobasal-A Medium supplemented with 2% B27 (Thermo Fisher Scientific, Karlsruhe, Germany), 1% N2 (Thermo Fisher Scientific, Karlsruhe, Germany), 1% PS, 0.001% CNTF (Merck, Darmstadt, Germany) and 0.001% BDNF (Merck, Darmstadt, Germany)]. The exchange to retinal cultivation media was evaluated before for toxicity to the RPE cells. The media of all RPE-monolayers, including the control monolayers without co-cultivation, was switched to retina cultivation media. To isolate retinal explants, first the cornea, lens and vitreous of the pig eyes were removed and a clover leaf-like structure was generated. By using a dermal punch (∅ = 3 or 8 mm, Pmf medical AG, Köln, Germany), the retinas were pierced in a circular manner as described previously^4^. In case of 8 mm punches, the eyecups were transferred into a petri-dish containing neurobasal medium (Neurobasal-A medium, Thermo Fisher Scientific, Karlsruhe, Germany) with 2% PS and the retinal explants were carefully removed with a spoon as the retina lifted from the RPE. By washing the explant in neurobasal media, remaining RPE cells were removed. Afterwards, the explant was placed in a trans-well insert (ThinCert, Greiner Bio-One, Frickenhausen, Germany) with or without a RPE monolayer with the ganglion layer (GCL) facing up^4^. For further cultivation of the retinal explants, one ml retina cultivation media in the well and 100 µl retina cultivation media in the insert was used. Explants were cultivated at 5% CO_2_ at 37 °C in the incubator. Controls were cultivated without RPE monolayers. Regarding the 3 mm explants, the retina was pierced in a circular manner with a 3 mm dermal punch. A spoon, moistened with 2% PS/Neurobasal medium, was carefully moved directly under the retinal punch to transfer the explant onto the spoon. Afterwards, the explant was washed once in a petri dish containing 2% PS/Neurobasal medium and transferred on a RPE monolayer of a 24 well plate Insert (ThinCert, Greiner Bio-One, Frickenhausen, Germany) with the GCL facing up^4^. Retina cultivation media was used for further cultivation (30 µl insert/500 µl well). For histological examinations, western blot and qRT-PCR, 8 mm retinal explants were used. For caspase 3/7 and cell viability assays, 3 mm explants were used. At the end of the indicated co-cultivation time, the explant was removed by aspirating and transferring it with a pipette tip. To remove RPE residues, the retinal explants were washed shortly in Neurobasal media. Immediately after this procedure, the analyses were performed or the monolayer was fixed.

### Trans-epithelial-resistance (TER)-measurements

TER measurements were performed on 12-well plate inserts with and without (blank) RPE-monolayers with a Millicell-ERS-2 m (Merck, Darmstadt, Germany) according to the manufacturer’s instructions. Briefly, a meter functionality test was always performed immediately before TER-measurements to ensure correct values. Monolayers were measured immediately after removing them from the incubator, to ensure similar temperature. The resistance was recorded in Ω, and the electrode was rinsed with culture media between different inserts. The blank resistance was calculated by determining the resistance of a trans-well insert containing only retinal culture media. The resistance of each RPE-monolayer was measured three times, and the mean value was determined. Afterwards, the blank was subtracted, and the value normalized to the measured area: TER (Ω* cm^2^). In case of 12 well plate Thincerts the growth area was 1.12 cm^2^.

### Cell viability, caspase 3/7 activity and ATP assay

To evaluate the cell viability of the RPE cells of the RPE-monolayers, CellTiter 96^®^ AQ_ueous_ One Solution Cell Proliferation Assay (MTS, Promega, Walldorf, Germany) was used according to the manufacturer's protocol. Briefly, 20 µl of MTS reagent was added to 100 µl retinal cultivation media in the Transwell—Insert (12-well plate). After 90 min, the media was transferred into a transparent 96-well plate (Greiner, Frickenhausen, Germany) and absorbance was measured (490/690 nm ratio) using a Tecan Reader (NanoQuant infinite M200) (Tecan, Männedorf, Switzerland).

CellTiter Glo 3D Assay (Promega, Walldorf, Germany) was used to evaluate ATP contents of the 3 mm retinal explants, according to the manufacturer’s protocol. Briefly, retinal explants were transferred in a white 96-well plate containing 50 µl retinal cultivation media. 50 µl CellTiter reagent was added; the plate was briefly mixed (using a plate shaker at 500 rpm for 30 s) and incubated at room temperature (RT) for 20 min. Afterwards, 100 µl of retinal cultivation media was added to each well to dilute the solution. In case of too high ATP levels, the solution was further diluted with retinal cultivation media (50 µl to 100 µl cultivation media) and luminescence was measured again with a luminometer (Tecan reader SPARK 10 M) (Tecan, Männedorf, Switzerland). The results are presented in relative light units [RLU]. The output values were multiplied by the dilution factor.

Activity of caspase 3/7 in RPE cells was determined using the lytic Caspase Glo 3/7 Assay from Promega (Promega, Walldorf, Germany), according to the manufacturer's protocols^80^. Briefly, 100 µl of caspase reagent was added to 100 µl of media in the inserts (12-well plate). After 1 h at RT, the cell solution was transferred to a white 96 well plate and luminescence was measured. The amount of luminescence was proportional to the amount of caspase activity in the sample. The results are presented in relative light units [RLU]. In case of retinal explants, 3 mm retinal explants were transferred to a white 96-well plate containing 100 µl of retinal cultivation media. 100 µl of caspase 3/7 Glo 3D Assay (Promega, Walldorf, Germany) was added and explants were briefly mixed using a plate shaker at 500 rpm for 30 s. Afterwards, explants were incubated for 30 min and luminescence was measured at a Tecan Reader.

### ELISAs

IL-1β as well as IFN-γ levels in the cultivation media of retinal explants (+ /− RPE-monolayers) were quantified using a Lumit Immunoassay (either for IL-1β or IFN-γ, Promega, Walldorf, Germany) according to the manufacturer’s protocol. Briefly, 50 µl of cultivation media was applied in duplicates into a white 96-well plate, together with the antibody conjugate (diluted 1:500). The plate was placed in an incubator (5% CO_2_ at 37° C) for 1 h. 20 µl of a 20-fold dilution of the detection substrate B in detection buffer B was added to each well. The luminescence was measured with a Tecan Spark (Tecan, Männedorf, Switzerland).

### Cytokine array

To semi-quantitatively investigate apoptotic protein expression, the porcine cytokine array C1 (AAP-CYT-1-2, Hölzel, Köln, Germany) was performed according to the manufacturer’s protocol. Briefly, media of five inserts was pooled (100 µl each) and 500 µl was used for each tested condition. Membranes were incubated with the media for 5 h at RT after blocking, followed by primary antibody incubation overnight. After the washing steps, incubation with the biotinylated antibody cocktail was performed for 1.5 h at RT. After a washing step, the membranes were treated with HRP-streptavidin for 2 h at RT, washed again, incubated with detection solution and the chemiluminescence signal was recorded (2 min, Odyssey Fc Imaging System, LI-COR, Bad Homburg vor der Höhe, Germany). The analysis was performed according to the manufacturer's instructions, followed by statistical processing with GraphPad Prism 9 (Graphpad software, San Diego, USA).

### Calcein-propidium iodide staining (live/dead staining)

After co-cultivation, RPE cells were stained with PI (1:100 in DPBS, 15 µM), Calcein (1 mM) and Hoechst (33324, 10 mg/mL in H_2_O stock solution diluted 1:2000 in PBS, ThermoFisher Scientific, Karlsruhe, Germany) for 15 min in the dark at RT. After washing with 1× PBS, cells were imaged in serum-free DMEM using a fluorescence microscope with AF555 (PI) (553/568 nm), GFP (Calcein) (488/509 nm) and DAPI (Hoechst) (350/461 nm) filter-sets.

### TUNEL (TdT-mediated dUTP-biotin nick end labeling) analysis

Retinal explants were cryo-protected using Tissue Tek (Sakura, Germany) and frozen in liquid nitrogen. Afterwards, explants were cut on a cryostat (12 μm sections) and fixed with 4% PFA for 20 min. After washing, sections were incubated in permeabilization solution [0.1% Triton X-100 (Sigma-Aldrich, Taufkirchen, Germany) in 0.1% Sodium citrate (Merck, Darmstadt, Germany)]. After another washing step, the positive control was treated with DNAse I (Sigma-Aldrich, Taufkirchen, Germany) for 10 min. Controls were washed again and all samples, except negative control, were incubated with 50 µl TUNEL reaction mixture as described by the manufacturer (Merck, Darmstadt, Germany). Negative controls were treated only with labelling solution. After 1 h at 5% CO_2_ at 37 °C in the incubator, all samples were washed and nuclei were stained with 5% DAPI/PBS (4',6-Diamidino-2-phenylindol, ThermoFisher Scientific, Karlsruhe, Germany) for 5 min. Pictures were taken using a fluorescent microscope (Axio Observer, Zeiss, Germany).

### Lipid-Green2 staining; autophagy staining

To investigate the deposition of neutral lipids, Lipidgreen2 staining (Sigma-Aldrich, Taufkirchen, Germany) was performed on (co-)cultivated RPE-monolayer. Briefly, retinal explants were carefully removed from co-cultivated RPE-monolayers without touching the RPE cells. The media of all RPE monolayers was removed completely and cells were washed with 1× PBS three times for 5 min. Afterwards, cells were covered with 10 µM Lipidgreen2 solution (in PBS) and incubated for 15 min in the incubator. After another washing step, the cells were fixed with 4% PFA/PBS for 15 min at RT and cells were washed again three times with PBS-T for 5 min. Nuclei were stained with DAPI. The membranes with the RPE monolayers were cut out of the inserts along the plastic well. On an object slide (Superfrost Plus slides, Carl Roth, Karlsruhe, Germany), the RPE-monolayers were placed in a drop of fluorsave (Merck, Darmstadt, Germany). The membranes were covered with another glass slide. Pictures were taken using fluorescence microscope (Axio Observer, Zeiss, Germany) with the ZEISS Software ZEN 3.5 (blue edition) (ZEISS, Germany).

Autophagy was evaluated with the Autophagy Assay Kit from Merck, Darmstadt, Germany (MAK138-1KT) according to the manufacturer’s protocol. Briefly, a working solution of the Autophagosome Detection Reagents was prepared by diluting the 500× solution in Stain Buffer, according to sample numbers. The retinal explants as well as the media was carefully removed and 100 µl of the autophagosome detection reagent working solution was added in every Insert. The cells were incubated at 37 °C with 5% CO_2_ for 1 h. Afterwards, the cells were washed with Wash Buffer three times by gently adding 200 µl of Wash Buffer to each Insert. The fluorescence intensity (λex = 360/λem = 520 nm) was determined with a fluorescence microscope (Axio Observer, Zeiss, Germany). Determination of positive cells was performed manually with ImageJ (National Institutes of Health, Bethesda, MD, USA). Mean fluorescence intensity was corrected to the background of each image and normalized to the cell number.

### Histological staining of the RPE monolayers

To investigate the patterns of tight junctions, ZO-1 expression of RPE-monolayers was evaluated. ZO-1 staining was performed as described previously^31^. To determine VEGFA expression, VEGFA antibody was used. Briefly, RPE-monolayers (after carefully removing the retinal explants), were fixated with 4% PFA/PBS. After washing with PBS-T, permeabilization solution (0.1% Triton-X-100/PBS) (Sigma-Aldrich, Taufkirchen, Germany) was added to each insert for 5 min at RT. The cells were washed again and blocked for 1 h in 5% BSA/PBS (Bovine serum albumin, Sigma-Aldrich, Taufkirchen, Germany) at RT. Afterwards, the primary ZO-1 antibody in 5% BSA/PBS or VEGFA was added directly to the cells and incubated overnight at 4 °C. After another washing step with PBS-T, the secondary antibody (in 5% BSA/PBS, Cat. Nr. 925-32219 for ZO-1 and 926-68070 for VEGFA) was added to the monolayers and incubated for 1 h RT. Cells were washed, and nuclei stained with DAPI. After another washing step, the trans-well membrane was removed from the inserts and placed into a drop of fluorsave on a microscope slide. The membranes were covered with another glass slide. Pictures were taken using a fluorescent microscope (Axio Observer, ZEISS, Germany) with the ZEISS Software ZEN 3.5 (blue edition) (ZEISS, Germany).

### qRT-PCR

Quantitative RT-PCR was performed as described before^81^. Briefly, the expression of inflammatory, cell death or cell markers (*IL-1β,* *IL-6,* *IL-8;* *Nf-kB,* *p62,* *TNF-α,* *Opsin,* *Rhodopsin*) were analysed. mRNA was isolated from the (co-cultured) 8 mm retinal explants and cDNA was reverse transcribed using the MultiMACs mRNA and cDNA Synthesis Kit on the MultiMACS™ M96 Separator (Miltenyi Biotec) according to the manufacturer’s protocol. After measuring the cDNA content, cDNA was diluted (1 ng/µl DNA was used) and qRT-PCR was performed (40 cycles), using Universal SYBR Green Supermix on a thermocycler (CFX96 Real-time-system, Biorad, Feldkirchen, Germany). The final primer solution-concentration was 100 nmol/L. cDNA expression levels of investigated genes were normalized to the expression of housekeeping genes (*RLP4* and *β-actin*). Primers (Table 2) were designed using the Primer3 software (GenBank: KM035791.1, http://www.bioinformatics.nl/cgi-bin/primer3plus/primer3plus.cgi/).

**Table 2:** qRT-PCR primer pairs in 5′–3′ directions.

Marker	Sequence forward (5′ → 3′)	Sequence reverse (5′ → 3′)
*β-Actin*	CACGCCATCCTGCGTCTGGA	AGCACCGTGTTGGCGTAGAG
*RPL4*	CAAGAGTAACTACAACCTTC	GAACTCTACGATGAATCTTC
*Beclin*	AGGAGCTGCCGTTGTACTGT	CACTGCCTCCTGTGTCTTCA
*Calbindin*	AGAATCCCACCTGCAATCAC	TGCCCATACTGATCCACAAA
*PKC-α*	ACCGAACAACAAGGAACGAC	CTGAGCTCCACGTTTCCTTC
*IL-8*	TGGCAGTTTTCCTGCTTTCT	CAGTGGGGTCCACTCTCAAT
*VEGF*	CTACCTCCACCATGCCAAGT	ACACTCCAGACCTTCGTCGT
*NF-kB*	AGGATGGGATCTGCACTGTC	ATCAGGGTGCACCAAAAGTC
*TNF-a*	CCACCAACGTTTTCCTCACT	CCAAAATAGACCTGCCCAGA
*Opsin*	GGGGAGCATCTTCACCTACA	GATGATGGTCTCTGCCAGGT
*Rhodopsin*	TCCAGGTACATCCCAGAAGG	GCTGCCCATAGCAGAAGAAG
*IL-6*	CACCAGGAACGAAAGAGAGC	GTTTTGTCCGGAGAGGTGAA
*IL-1β*	CCAAAGAGGGACATGGAGAA	TTATATCTTGGCGGCCTTTG
*TLR3*	GGTACTGTTGCCCTTTTGGA	AATTCTGGCTCCAGCTTTGA

The listed primer pairs were used in qRT-PCR experiments, while *β-actin* and *RLP4* were used as housekeeping genes.

### Western-blot

10% Mini-PROTEAN TGX Precast Gels (Bio-rad, Feldkirchen, Germany) were used according to manufacturer’s protocol (https://www.bio-rad.com/de-de/category/western-blotting?ID=9324fd3c-6af4-4551-831e-8db6fa3f3452). Briefly, 15 µg protein was loaded with a total volume of 10 µl per lane (containing 1× Laemmli Buffer, Bio-rad, Feldkirchen, Germany). After electrophoresis (PowerPac HC High-current Power Supply (Bio-Rad, Feldkirchen, Germany), 100 V), transfer was performed (Cytiva Amersham Protran NC-Membrane, 0.45 µM, Fisher-Scientific, Schwerte, Germany). Transfer-Buffer was Towbin, containing 25 mM Trizma (Merck, Darmstadt, Germany), 192 mM Glycin (Merck, Darmstadt, Germany) and 20% Methanol. Transfer was performed at 200 mA for 2,5 h. Ponceau-staining confirmed successful transfer. After blocking with EveryBlot Blocking buffer (Bio-rad, Feldkirchen, Germany) for 10 min, immunostaining was performed using antibody against VEGFA (Table 1) and β-actin for each sample. Secondary antibody IRDye 800 RD goat anti-mouse (Table 1) against VEGFA and IRDye 680 RD goat anti-rabbit against β-Actin was used to visualize protein bands. All antibodies were diluted in EveryBlot Blocking buffer containing 0.05% Tween-20. Protein bands were recorded at 700 and 800 nm and evaluated with the Odyssey infrared imager system 2.1 (LI-COR Bioscience). VEGFA (46 kDA) signal intensities were normalized to β-actin (42 kDa) signal intensities.

### Image generation

Overview image was generated using the platform of https://www.biorender.com.

### Statistical analysis

All results are presented as mean ± SEM. Statistical analysis was performed using GraphPad PRISM 9. Normality was evaluated using Shapiro–Wilk test and Kolmogorov–Smirnov test. In case of normal distribution, ANOVA (more than 2 comparisons) or t-test (two comparisons) was used. Welch’s tests were used when variance and/or sample size between groups differed. Non-parametric tests were used when datasets weren’t normal (Mann–Whitney and Kruskal–Wallis). Differences were significant at p < 0.05. Statistical differences are indicated as * with p < 0.05, ** with p < 0.01, *** p < 0.001 and **** p < 0.0001 compared to the control.

### Supplementary Information

Supplementary Figures.

## Data Availability

The data that support the findings of this study are available from the corresponding author, [Dr. Sven Schnichels], upon reasonable request.
