# Supplementary material for: Co-cultivation of primary porcine RPE cells and neuroretina induces inflammation: a potential inflammatory AMD-model
Source: Sci Rep. 2023 Nov 7;13:19345. doi: 10.1038/s41598-023-46029-8 (PMC10630302; doi:10.1038/s41598-023-46029-8)
Supplement: Supplementary file 1 — Supplementary Figures. [file 41598_2023_46029_MOESM1_ESM.docx]

**Co-cultivation of primary RPE cells and retina induces inflammation: A potential inflammatory AMD-model**

**Agnes Fietz ^1^, Sven Schnichels ^1*^, Jose Hurst ^1^**

^1^ Centre for Ophthalmology, University Eye Hospital Tübingen, 72076 Tübingen, Germany

*Correspondence: [sven.schnichels@med-tuebingen.de](mailto:sven.schnichels@med-tuebingen.de)


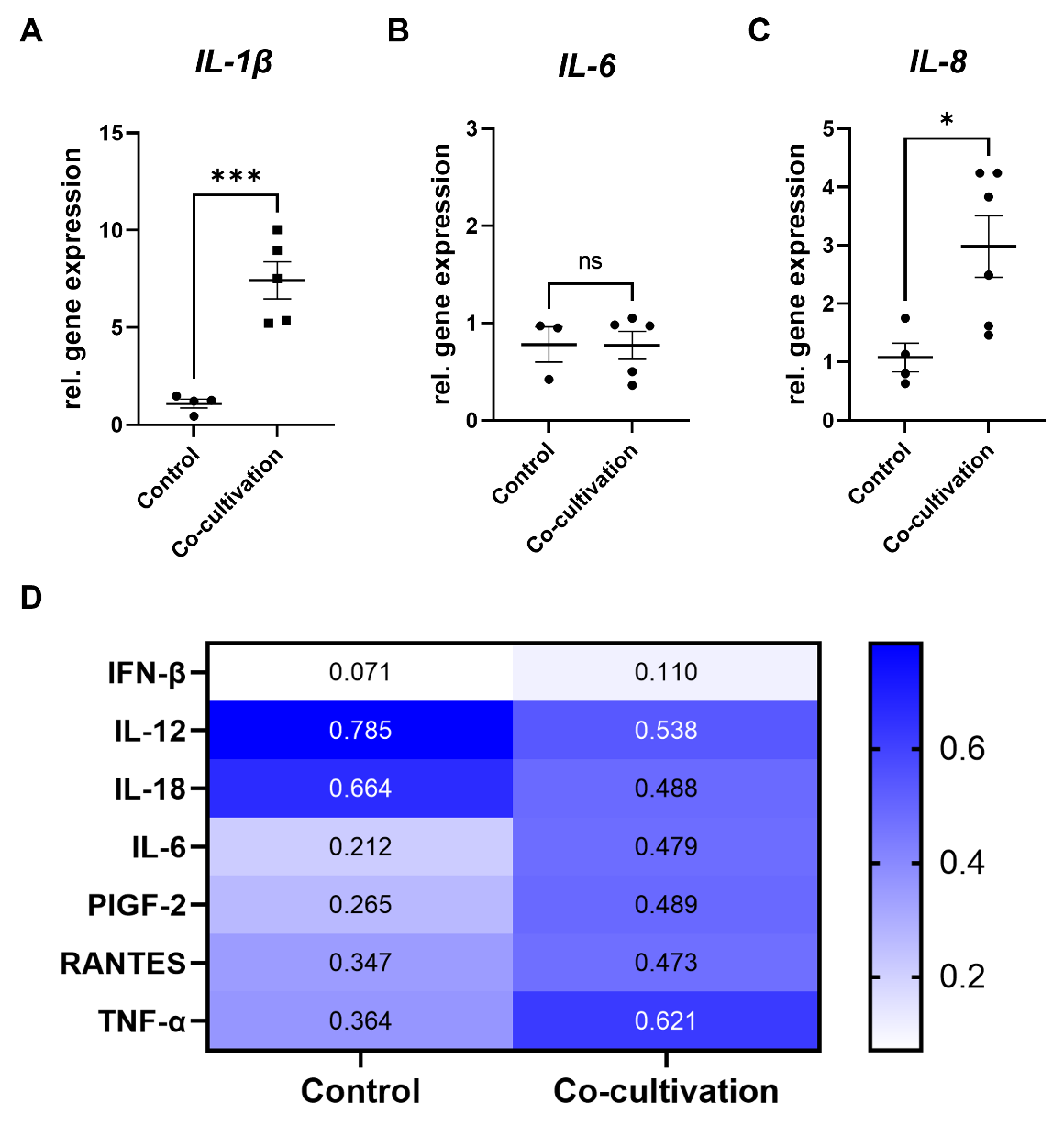


**Supplemental Figure 1: Co-cultivation induced inflammatory reaction after 24 h in the retinal explant**

Porcine retinal explants were directly co-cultivated with RPE-monolayers for 24 h. **A** Expression of pro-inflammatory *IL-1β* was significantly enhanced due to co-cultivation. **B** In contrast, 24 h co-cultivation did not increase *IL-6* expression in retinal explants. **C** Angiogenesis marker, pro-inflammatory cytokine *IL-8* was significantly induced in co-cultivated retinal explants. Students t-test. * With p<0.05, *** p<0.001. **D** Supernatant of 5 retinal explants or co-cultivated retinal explants was collected, and a semi-quantitative Cytokine array was performed. Protein expression of pro-inflammatory cytokines (IL-6, TNF-α) was upregulated, in contrast to anti-inflammatory and pleiotropic cytokines (IL-12, IL-18). Co-cultivation furthermore resulted in increased IFN-β and PIGF-2 expression.

**
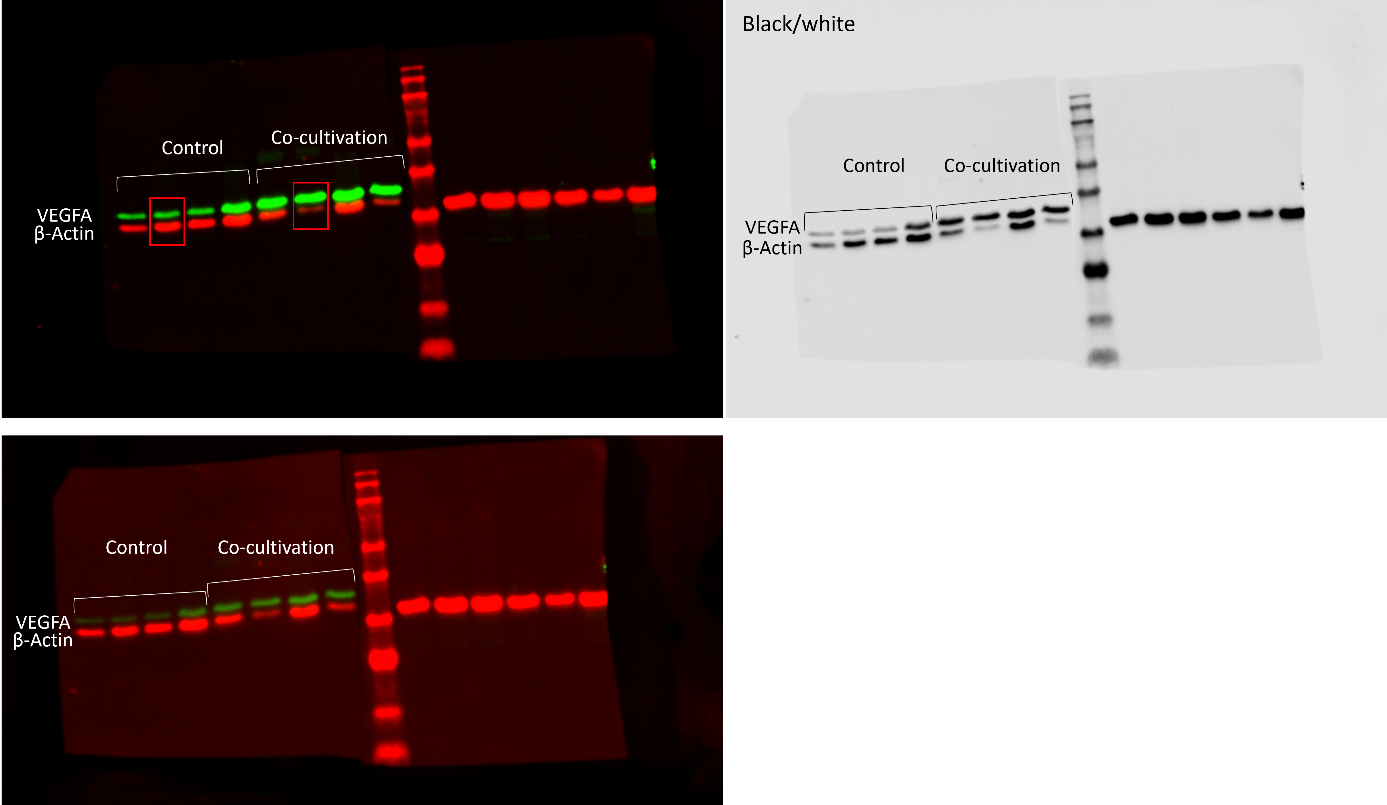
**

**Supplemental Figure 2: Original Western-blot of the VEGFA expression in (co-)cultivated retinal explants**

Porcine retinal explants were directly co-cultivated with RPE-monolayers for 48 h. 15 µg protein was loaded with a total volume of 10 µl per lane. Immunostaining was performed using antibodies against VEGFA and β-actin for each sample. Protein bands were recorded at 700 and 800 nm and evaluated with the Odyssey infrared imager system. VEGFA (46 kDA) signal intensities were normalized to β-actin (42 kDa) signal intensities. Pictures of different contrast intensities and a black/white picture are shown to demonstrate gel borders. Red box: cropped image for Figure 1G. N=4.


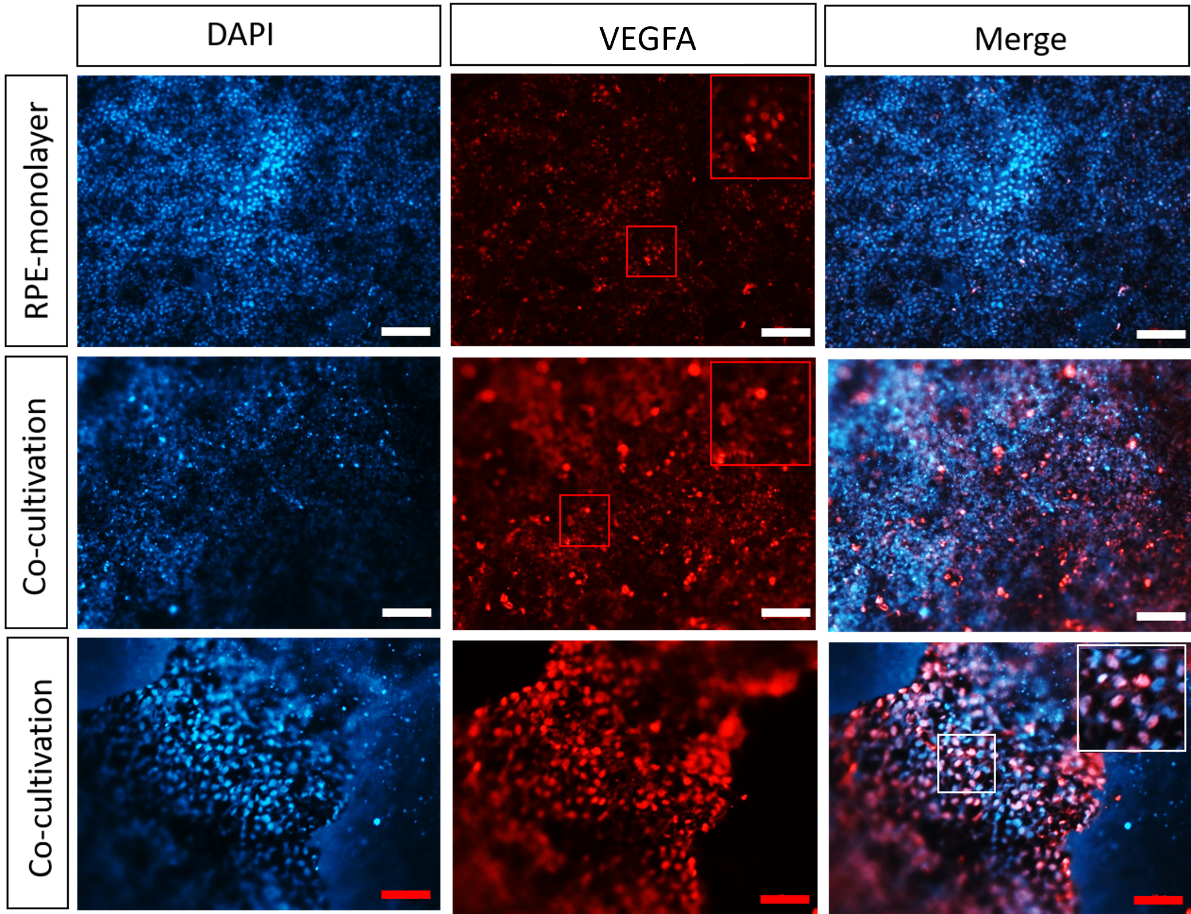


**Supplemental Figure 3: Co-cultivation led to enhanced expression of angiogenesis marker VEGF-A in RPE-monolayers.**

RPE-monolayers were directly co-cultivated with porcine retinal explants for 48 h. VEGF-A expression was strongly increased in co-cultivated RPE-monolayers. Representative pictures, N=4. White scale bar: 100 µM. Red scale bar: 50 µM.


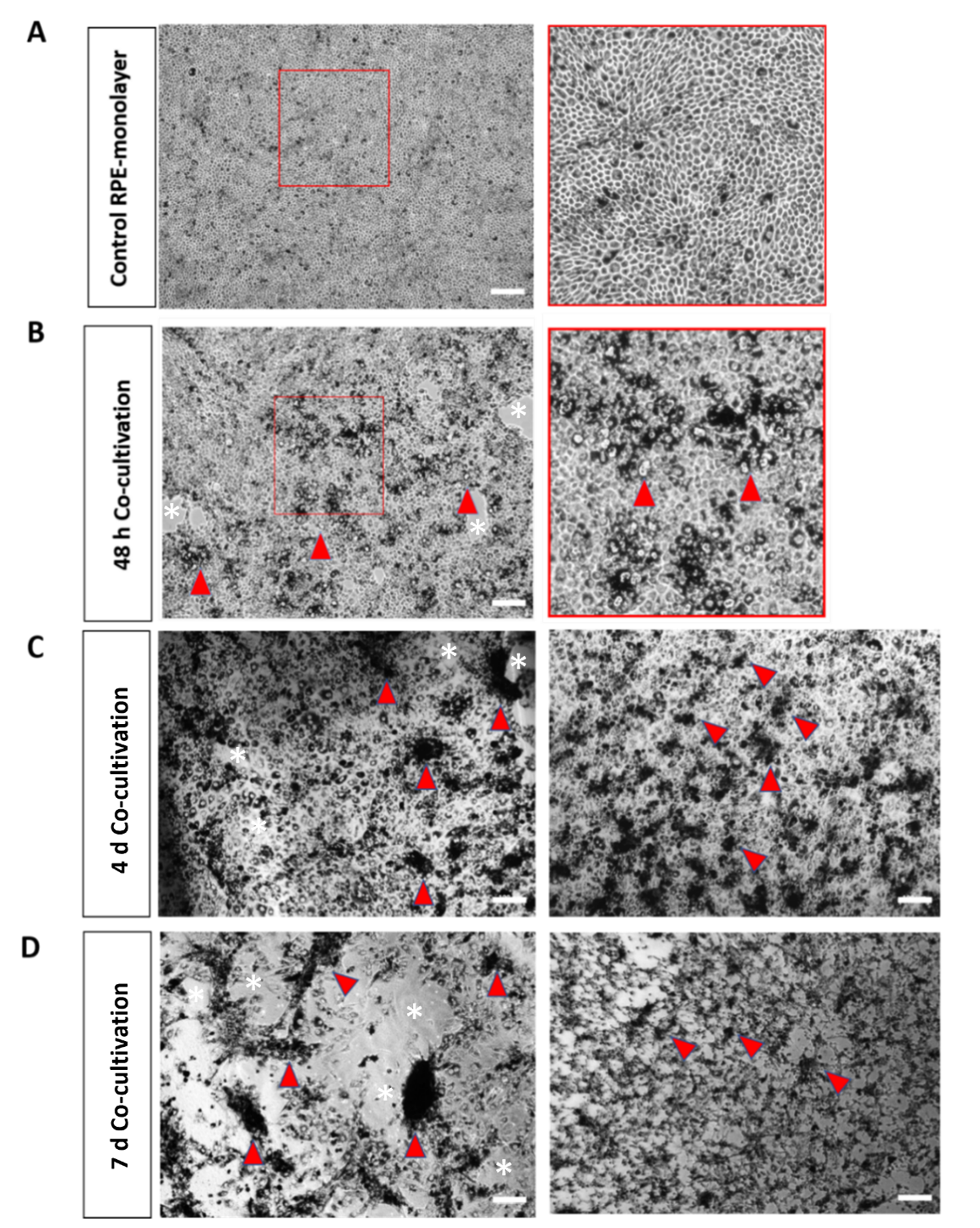


**Supplemental Figure 4 Direct co-cultivation resulted in disruption of the RPE-monolayer**

Primary RPE-monolayer (**A**) were directly co-cultivated with porcine retinal explants for **B** 48 h, **D** 4 d and **D** 7 d. Morphology was investigated by phase-contrast microscopy. Representative brightfield images are shown. **A** Confluent RPE-monolayers without co-cultivation demonstrate normal hexagonal RPE morphology and homogenous pigmentation. **B** 48 h after direct co-cultivation, dead RPE cells (white stars), as well as hyperpigmentation and accumulated pigment, occurred (red arrows). First disruptions in the RPE-monolayers were detectable (white stars). **C** Direct co-cultivation for 4 d led to clumps of dead RPE cells, seen as an accumulation of pigment in the RPE-monolayer (red arrows). Also, more disruptions of the monolayer (white stars) were observable. **D** 7d of co-cultivation resulted in a strong decrease in RPE cell number, as seen in cell-free areas and clumps of highly pigmented RPE cells (red arrows). Almost all RPE cells were dead. Abbreviations: d=days; h= hours. Scale bar: 200 µM. Representative pictures are shown, n=4.


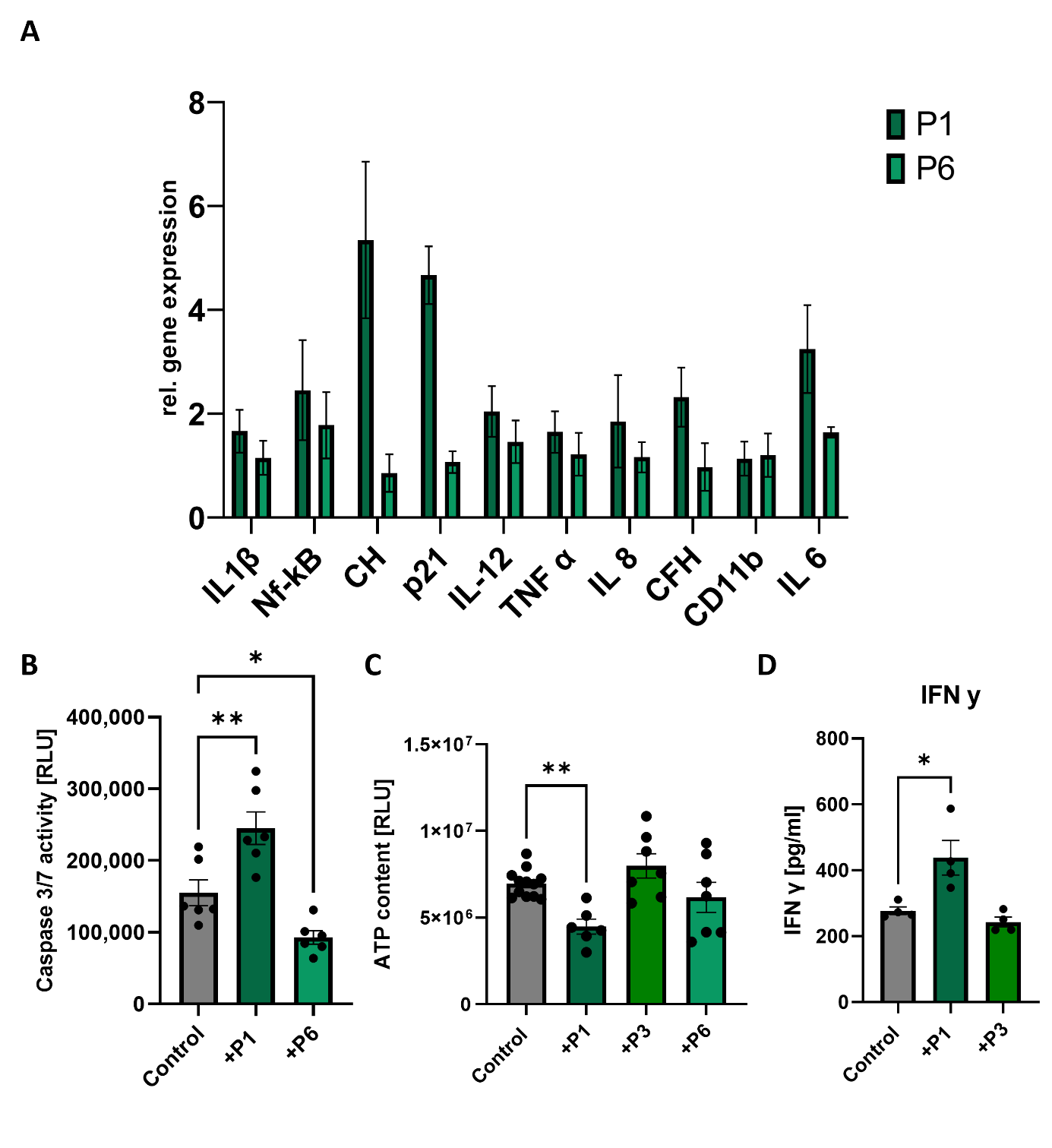


**Supplemental Figure 5: Co-cultivation with higher RPE-Passages resulted in less inflammation and cell death**

RPE-monolayers containing RPE cells from Passages 1-6 were co-cultivated with porcine retinal explants for 48 h. **A** Compared to co-cultivated RPE-P1-monolayer, co-cultivated RPE-P6-monolayer demonstrated decreased pro-inflammatory and pro-cell death gene expression. Relative gene expression is shown compared to not cultivated RPE-monolayers. n=4. **B** Induction of cell death was quantified by caspase 3/7 activity. Co-cultivation with RPE-P1-monolayers led to a significant increase of caspase 3/7 and thus apoptosis. In contrast, co-cultivation with a P6-monolayer decreased caspase 3/7 activity. n=6, ANOVA. The experiment was repeated three times with similar results. **C** ATP levels did only drop in retinal explants which were co-cultivated with RPE-P1-Monolayers. n=6, repeated three times with similar results. Welch’s-ANOVA. **D** IFN γ expression was only increased due to co-cultivation with RPE-P1-monolayers. n=4, ANOVA. Mean ± SEM is shown. **p<0.05; **p<0.01; ***p<0.001; ****p<0.0001.*
